# Revision of Taiwanese species of *Atrachya* Chevrolat, 1836 (Coleoptera, Chrysomelidae, Galerucinae): descriptions of three new genera, two new species, and designations of three new synonyms

**DOI:** 10.3897/zookeys.940.51800

**Published:** 2020-06-11

**Authors:** Chi-Feng Lee

**Affiliations:** 1 Applied Zoology Division, Taiwan Agricultural Research Institute, Taichung 413, Taiwan Taiwan Agricultural Research Institute Taichung Taiwan

**Keywords:** *
Chinochya
*, leaf beetles, *
Neochya
*, new genus, taxonomic revision, *
Tsouchya
*

## Abstract

The genus *Atrachya* Chevrolat is redefined based on study of the type species *A. menetriesii* (Faldermann, 1835). All Taiwanese species of *Atrachya* are transferred to three new genera: *A. hirashimai* Kimoto, 1976 and *A. nitidissima* (Chûjô, 1935) are transferred to *Neochya***gen. nov.**; *A. mediofasciata* Kimoto, 1976 is transferred to *Tsouchya***gen. nov.**; *A. unifasciata* Takizawa, 1978 is transferred to *Chinochya***gen. nov**. Two species are described: *N. chengi***sp. nov.** and *N. tsoui***sp. nov.***Atrachya bicoloripennis* (Chûjô, 1938) and *A. saramao* (Chûjô, 1962) are regarded as synonyms of *N. nitidissima* (Chûjô, 1935) **comb. nov.**, and *Monolepta tsoui* Lee, 2009 is synonymized with *T. mediofasciata* (Kimoto, 1976) **comb. nov.***Monolepta sublata* Gressitt & Kimoto, 1963 is redescribed and transferred to *Chinochya***gen. nov.** Taiwanese records of *Monolepta sublata* are based on misidentifications and represent specimens of *C. unifasciata*. Variablity of adult color patterns is discussed.

## Introduction

The genus *Atrachya* Chevrolat, 1836 is attributed to “Monoleptites” *sensu*Wilcox (1973) having elongate metatarsomere I. Twenty-seven species of *Atrachya* were documented by Nie et al. (2017). Besides *Atrachya*, *Paleosepharia* Laboissière, 1936 and *Monolepta* Chevrolat, 1836 are members of this group recorded from Taiwan. Chûjô (1935) described the first species as *Luperodes nitidissimus* from Taiwan. Later, he described two additional species, *L. bicoloripennis* Chûjô, 1938 and *L. saramao* Chûjô, 1962. Kimoto (1969) transferred these Taiwanese species of *Luperodes* to *Atrachya* and described a fourth species, *A. hirashimai* and later, a fifth, *A. mediofasciata* Kimoto, 1976. The last, sixth species, was described as *A. unifasciata* by Takizawa (1978).

Diagnostic characters of the genus *Atrachya* were discussed (Wagner and Bieneck 2017) based on *Cnecodes bisignatus* Motschulsky, 1858. However, one important character, the sexually dimorphic elytral impression, does not occur in any Taiwanese species. This paper redefines the genus *Atrachya* based on the type species, *A. menetriesii* (Faldermann, 1835). The taxonomic status of the Taiwanese species of *Atrachya* is reevaluated, and their identities reviewed. In addition, Kimoto (1976) recorded *Monolepta sublata* from Taiwan, which is extremely similar to *A. unifasciata* Takizawa, 1978. These two species are compared to clarify their taxonomic status.

**Figure 1.**
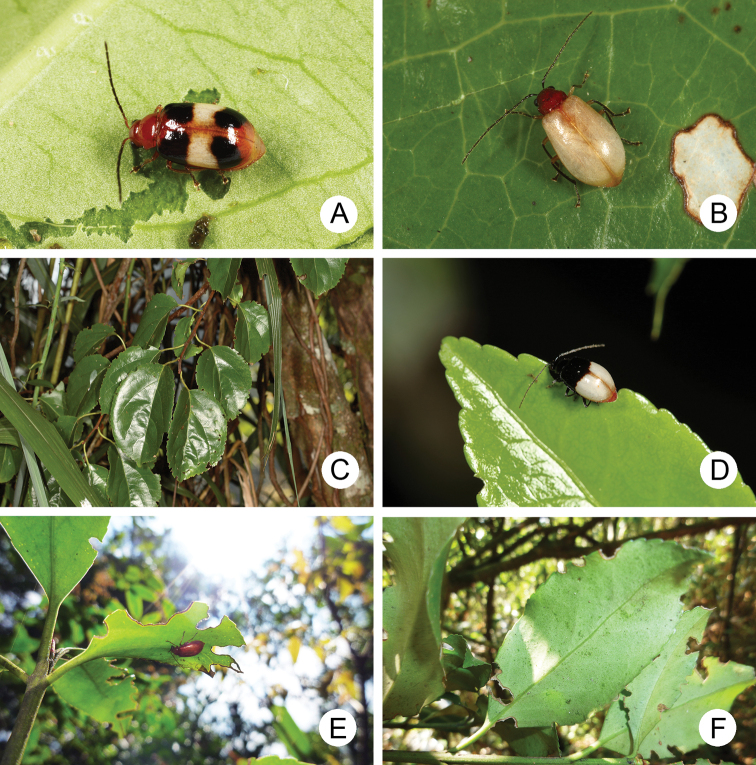
Habitat photographs **A** adult of *Neochya chengi* sp. nov. feeding on leaves of *Celastrus hindsii***B** adult of *N. hirashimai***C***Celastrus kusanoi*, food plant for *Neochya* species **D** adult of *N. nitidissima* feeding on leaves of *Euonymus spraguei***E** adult of *N. tsoui* resting on underside of leaves of *Euonymus spraguei***F***Euonymus spraguei*, food plant for *Neochya* species.

The Taiwan Chrysomelid Research Team (TCRT) has been inventorying chrysomelid fauna since 2005. Adults of the taxa covered in this paper were collected by sweeping host plants, species of *Celastrus* and *Euonymus* (Celastraceae) (Fig. 1). More than 600 specimens were collected using this method in addition to loaned specimens, providing an adequate sample set for assessing species diversity of the group.

## Materials and methods

The abdomens of adults were separated from the bodies and boiled in 10% KOH solution, followed by washing in distilled water to clear and soften genitalia. The genitalia were then dissected from the abdomen, mounted on slides in glycerin, and studied and drawn using a Leica M165 stereomicroscope. A Nikon ECLIPSE 50i microscope was used for detailed examination.

At least two pairs from each species were examined to delimit variability of diagnostic characters. For species collected from more than one locality, at least one pair from each locality was examined. Length was measured from the anterior margin of the eye to the elytral apex, and width at the greatest width of the elytra. Descriptions are all based on adult specimens.

Specimens were available for study and deposited in the following institutions:

**BPBM**Bernice P. Bishop Museum, Hawaii, USA [James Boone];

**CAS**California Academy of Sciences, California, USA [David H. Kavanaugh];

**KMNH**Kitakyushu Museum of Natural History and Human History, Kitakyushu, Japan [Yusuke Minoshima];

**KUEC**Faculty of Agriculture, Kyushu University, Fukuoka, Japan [Osamu Tadauchi];

**NMNS** National Museum of Natural Science, Taichung, Taiwan [Jing-Fu Tsai];

**OMNH**The Osaka Museum of Natural History, Osaka, Japan [Shigehiko Shiyake];

**SDEI**Senckenberg Deutsches Entomologisches Institut, Muncheberg, Germany [Mei-Ling Chan, Jing-Fu Tsai];

**SEHU**Systematic Entomology, The Hokkaido University Museum, Sapporo, Japan [Masahiro Ohara];

**TARI**Taiwan Agricultural Research Institute, Taichung, Taiwan..

Exact label data are cited for all type specimens of previously described species; a double slash (//) divides the data on different labels and a single slash (/) divides the data in different rows. Other comments and remarks are in square brackets: [p] – preceding data are printed, [h] – preceding data are handwritten, [w] – white label, [y] – yellow label, [r] – red label, [b] – blue label.

## Taxonomy

### Atrachya

Taxon classificationAnimaliaColeopteraChrysomelidae

Chevrolat, 1836

772BCBB6-4B55-5724-8660-C6BF16DA7378

Atrachya Chevrolat, 1836: 401 (type species: Galleruca menetriesii Faldermann, 1835, by monotypy).
Iphidea Baly, 1865: 127 (type species: Iphidea discrepens Baly, 1865, by original designation) (= Galleruca menetriesii Faldermann, 1835. Synonymized by Gressitt and Kimoto (1963)).
Cnecodes Motschulsky, 1858: 99 (type species: Cnecodes bisignatus Motschulsky, 1858, by Weise (1892)) (= Chrysomela bimaculata Hornested, 1788. Synonymized by Weise (1892)). Synonymized by Wagner and Bieneck (2012).

#### Examined specimens of *Atrachya menetriesii*

**(Faldermann, 1835).** Japan. Hiroshima: 1♂ (TARI), Mihara-shi, Yahata-cho, Honjo, 28.VI.2013, leg. H. Suenaga; Hokkaido: 1♂ (TARI), Sapporo-shi, Minami-ku, Kannonzawa, 26.VII.2011, leg. H. Suenaga; Okayama: 1♀ (TARI), Maniwa-shi, Hiruzen, Kamitokuyama, 7.VII.2007, leg. H. Suenaga; 1♀ (TARI), Maniwa-shi, Hiruzen, Utsumi-toge, 20.VII.2013, leg. H. Suenaga; Tokushima: 1♀ (TARI), Minokoshi, Tsurugisan, Miyoshi-shi, 22.VII.2007, leg. S. Sejima; Tottori: 1♂ (TARI), Hoki-cho, Iwatate, Masunizu-kogen, 20.VII.2013, leg. H. Suenaga.

#### Remarks.

**Figure 2.**
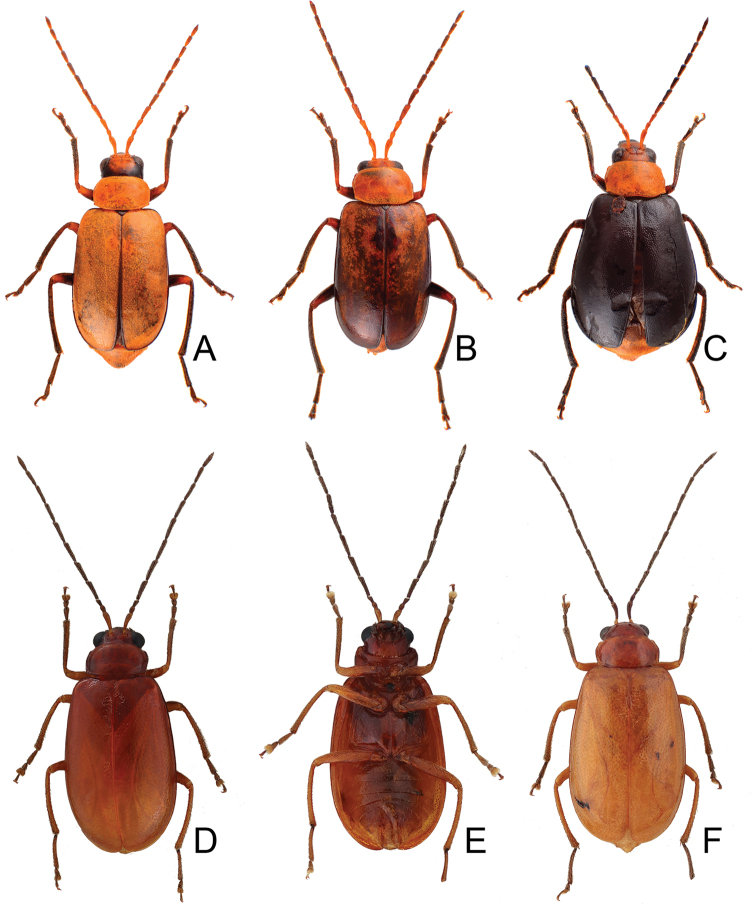
Habitus of *Atrachya menetriesii* and *Neochya tsoui***A***A. menetresii*, male, dorsal view **B** same, male, color variation **C** same, female, dorsal view **D***N. tsoui*, male, dorsal view **E** same, ventral view **F** same, female, dorsal view.

**Figure 3.**
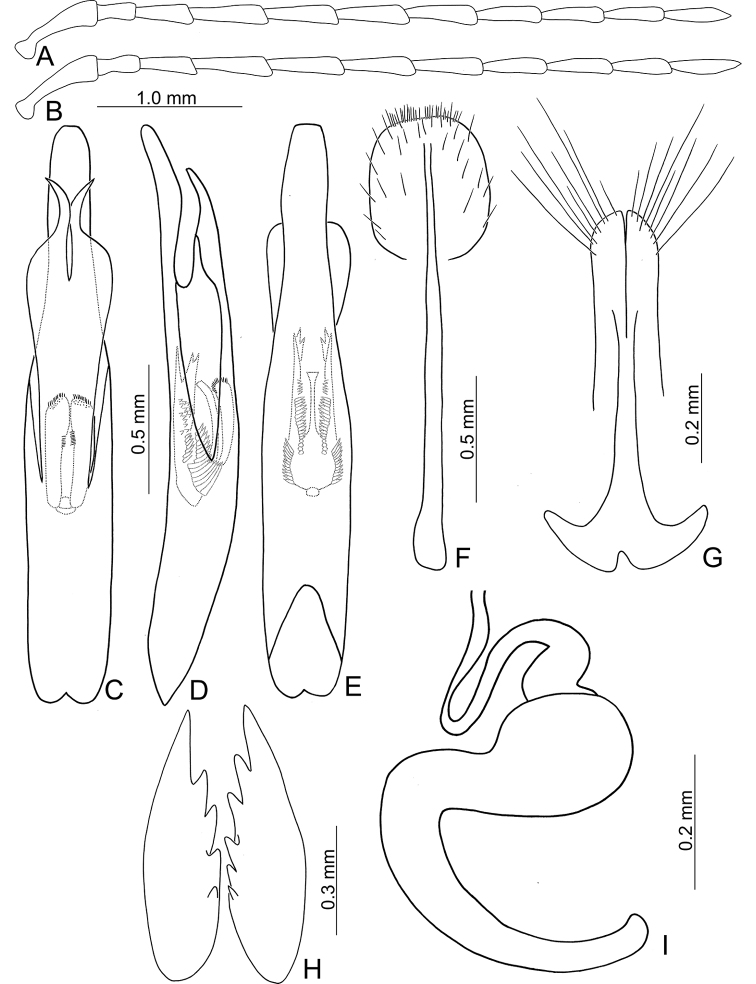
Diagnostic characters of *Atrachya menetriesii***A** antenna, male **B** antenna, female **C** aedeagus, dorsal view **D** aedeagus, lateral view **E** aedeagus, ventral view **F** abdominal ventrite VIII **G** gonocoxae **H** bursal sclerites **I** spermatheca.

*Atrachya* is a distinct genus similar to *Paleosepharia* Laboissière (redefined based on type species by Rizki et al. (2016) and Taiwanese species by Lee (2018)) in possessing elongate antennomere III that is much longer than antennomere II (Fig. 3A, B) (both antennomeres subequal in length in *Monolepta*), the presence of a subscutellar impression or groove on the elytra in males (Fig. 2A–C) (absent in those of *Monolepta*). In addition, females of *Atrachya* share some genitalic characters with *Paleosepharia*, including only one pair of bursal sclerites (Fig. 3H), similar shapes of spermatheca (Fig. 3I) and gonocoxae (Fig. 3G). However, members of *Atrachya* differ from those of *Paleosepharia* in having uniform tarsomere I of front legs (sexual dimorphic tarsomere I of front legs in *Paleosepharia*), almost straight apex of penis in lateral view (Fig. 3D) (dorsally curved apex of penis in lateral view in *Paleosepharia*), deeply incised tectum with strong apical hooks (Fig. 3C) (apical tapering tectum or weakly incised tectum without apical hooks in *Paleosepharia*).

#### Included species.

Excluding Taiwanese species, 21 species in the African, Palaearctic, and Oriental regions (Nie et al. 2017). Taxonomic status of species should be reevaluated (see below).

### Neochya gen. nov.

Taxon classificationAnimaliaColeopteraChrysomelidae

400FC28B-AA17-5C01-AF26-64A28FF7AA98

http://zoobank.org/A10C4F16-751F-4EC1-B4F0-655AA94D9208

#### Type species.

*Atrachya hirashimai* Kimoto, 1969

#### Description.

***Coloration***: extremely variable but without metallic color. Body length 4.6–7.0 mm.

***Head*.** Labrum trapezoidal, transverse, with six pores in transverse row bearing pale setae, anterior margin medially depressed. Anterior part of head very short, almost impunctate and glabrous, several setae on anterior margin of clypeus and anterofrontal ridge. Interantennal space broad, 1.5–2.6× as wide as diameter of antennal insertion. Frontal tubercles transverse, subtriangular, slightly elevated, glabrous. Vertex smooth and glabrous. Antennae slender, covered with dense setae, antennomere II as long as antennomere III; similar in both sexes.

***Pronotum*** 1.75–2.00 times as broad as long, lateral margins slightly rounded, basally narrowed. Disc covered with dense, coarse punctures, moderately or strongly convex, with lateral depressions, except *N. chengi* sp. nov. and *N. nitidissima*. Anterior margin lacking marginal bead, lateral and posterior margins with marginal bead. Anterior and posterior margins without setae, lateral margins with two pairs of setae near base and apex respectively. Anterior angles moderately swollen, rectangular, posterior angles obtuse angulate, all angles with setigerous pore bearing long pale seta.

***Scutellum*** subtriangular, impunctate, glabrous, with rounded apex.

***Elytra*** ca 1.35–1.68 times as long as wide, almost glabrous (with indistinct, sparse, short, pale setae on humeri, lateral margins and apical slopes), parallel sided, except *N. nitidissima* (Fig. 9, broader in middle), covered with densely confused punctures. Humeral calli well developed. Epipleura broad at base, gradually narrowed from basal 1/3, abbreviated near apex (Fig. 19D). Macropterous.

***Ventral*** surface sparsely covered with fine punctures and pale setae. Anterior coxal cavities widely open (Fig. 18D). Prosternal process not visible between procoxae. Abdomen simple, posterior margin of last ventrite with two long incisions in males.

***Legs slender*.** All tibiae with one apical spine, the longest spine on metatibia. Protarsomeres I not modified in males. Metatarsomeres I much longer than pro- and mesotarsomeres I, much longer than II and III combined. Claws appendiculate.

***Penis*** broad, with one pair of small lateral processes near apex (Figs 8C–E, 10C–E, 12C–E) (except *N. chengi* sp. nov. (Fig. 5C–E)); tectum broad, apical margin truncate; internal sac with only one type of endophallic spiculae (median endophallic spiculae).

***Gonocoxae*** (Figs 5F, 8F, 10F, 12F) slender, tightly conjunct medially; each gonocoxa with eight setae from near apex to apical 1/6, subapically widened, apex narrowly rounded, base bifurcate. Ventrite VIII (Figs 5G, 8G, 10G, 12G) weakly sclerotized except apex, with several short and long setae at apex, and several long setae at sides, spiculum elongate. Spermathecal receptaculum (Figs 5H, 8H, 10H, 12H) as slender as pump, apically tapering; pump slender and curved; sclerotized spermathecal duct extremely elongate, but base wide, followed by short slender tube with inflated areas. Bursal sclerites reduced.

#### Diagnosis.

*Neochya* gen. nov. differs from *Atrachya* Chevrolat and *Monolepta* Chevrolat in the following combination of characters: antennomere II subequal to III in length (antennomere II much shorter than III in *Atrachya* (Fig. 3A, B)); widely open prothoracic coxal cavities (Fig. 18D) (closed prothoracic coxal cavities in Taiwanese species of *Monolepta*, Fig. 18C); absence of subscutellar impression on the elytra in males (presence of subscutellar impression on the elytra of *Atrachya*); penis with tectum broad and apical margin truncate (Figs 5C, 8C, 10C, 12C) (tectum elongate with apex deeply bifurcate in *Atrachya* (Fig. 3C)), only one type of endophallic spiculae (Figs 5C–E, 8C–E, 10C–E, 12C–E) (three types of endophallic spiculae in *Monolepta*); gonocoxae slender and subapically broadened (Figs 5F, 8F, 10F, 12F) (gonocoxae broad and parallel-sided in *Atrachya* (Fig. 3F)), spermathecae with slender receptaculum as pump (Figs 5H, 8H, 10H, 12H) (greatly swollen receptaculum in *Atrachya* (Fig. 3I)), with apex acute (without acute apex in *Atrachya*); reduced bursal sclerites (well-developed bursal sclerites in *Atrachya* (one pair (Fig. 3H)) and *Monolepta* (two pairs); ventrite VIII with few lateral setae (Figs 5G, 8G, 10G, 12G) (dense lateral setae in *Atrachya* (Fig. 3F)).

#### Etymology.

Composed from new and *Atrachya* to indicate that this is a new genus similar to *Atrachya*.

#### Included species.

*Neochya chengi* sp. nov., *N. hirashimai* (Kimoto), comb. nov., *N. nitidissima* (Chûjô) comb. nov., and *N. tsoui* sp. nov.

### Neochya chengi sp. nov.

Taxon classificationAnimaliaColeopteraChrysomelidae

2B0EA8FD-CFDF-5AC5-A623-7AB6782ACCF9

http://zoobank.org/8B7C01A7-66F1-42AB-B0FD-48833F2D5D96

Figures 4 , 5

#### Types

**(*N* = 49). *Holotype*** ♂ (TARI): Taiwan. **Pingtung**: Tahanshan (大漢山), 30.III.2015, leg. I.-L. Lee. ***Paratypes*.** 3♂♂ (TARI), same locality, 7.II.2008, leg. M.-H. Tsau (= Tsou); 1♀ (TARI), same locality, 6.II.2008, leg. S.-F. Yu; 1♀ (TARI), same locality, 6.II.2008, leg. M.-H. Tsou; 1♀ (TARI), same locality, 3.III.2008, leg. C.-F. Lee; 3♂♂, 2♀♀ (TARI), same locality, 22.I.2009, leg. M.-H. Tsou; 1♂, 2♀♀ (TARI), same locality, 24.I.2009, leg. M.-H. Tsou; 1♂, 2♀♀ (TARI), same locality, 21.III.2009, leg. M.-H. Tsou; 2♂♂, 1♀ (TARI), same locality, 5.IV.2009, leg. C.-F. Lee; 1♂, 1♀ (TARI), 15.II.2010, leg. M.-H. Tsou; 1♀ (TARI), same locality, 6.I.2012, leg. Y.-T. Chung; 1♀ (TARI), Chunri (春日), 5.IV.2015, leg. J.-C. Chen; 1♀ (TARI), Kenting (墾丁), 23.VIII.2016, leg. Y.-T. Chung; 2♀♀ (TARI), Lilungshan (里龍山), 10.XI.2009, leg. J.-C. Chen; 1♀ (TARI), same locality, 23.XII.2009, leg. J.-C. Chen; 1♂ (TARI), same locality, 2.III.2012, leg. J.-C. Chen; 2♂♂ (TARI), Nanjenhu (南仁湖), 31.III.2011, leg. J.-C. Chen; 1♂, 2♀♀ (TARI), Shouka (壽卡), 5.II.2008, leg. S.-F. Yu; **Hsinchu**: 1♀ (TARI), Lupi (魯壁), 20.VII.2008, leg. S.-F. Yu; 1♂ (TARI), same locality, 10.III.2009, leg. H. Lee; **Taitung**: 1♀ (TARI), Imalintao (依麻林道), 4.II.2008, leg. M.-H. Tsou; **Yunlin**: 1♂, 11♀♀ (TARI), Chiananyunfeng (嘉南雲峰), 29.IX.2013, leg. W.-C. Liao.

#### Description.

**Figure 4.**
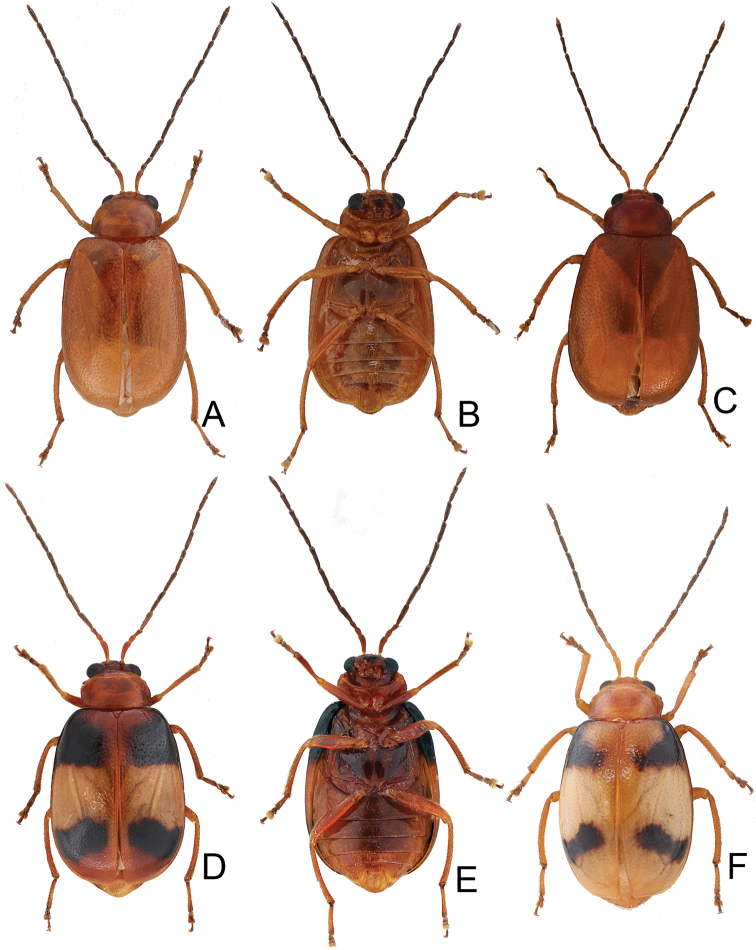
Habitus of *Neochya chengi* sp. nov. **A** male, dorsal view **B** same, color variation **C** female, dorsal view **D** male, from Tahanshan (大漢山), dorsal view **E** same, ventral view **F** female, from Kenting (墾丁), dorsal view.

**Figure 5.**
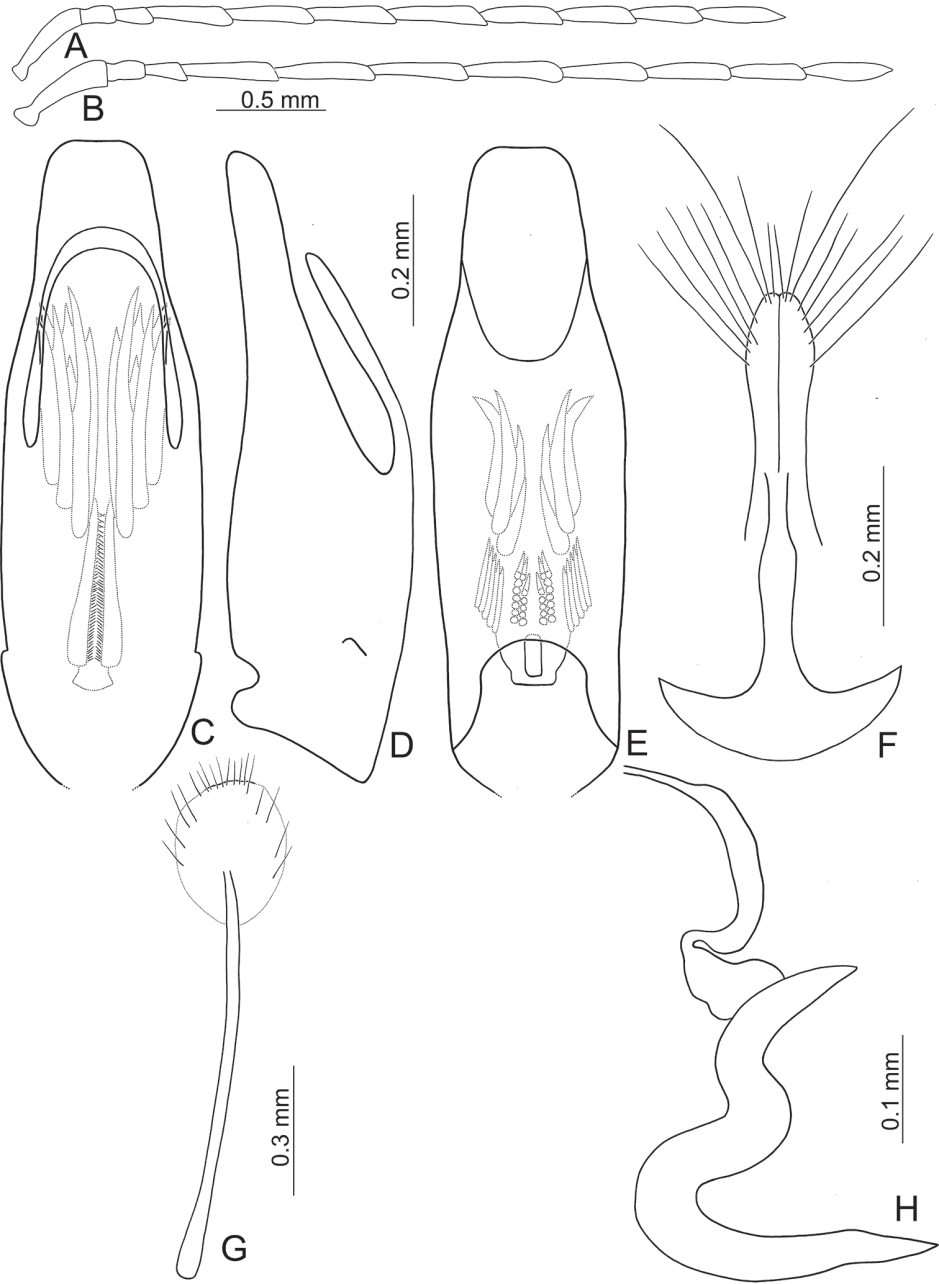
Diagnostic characters of *Neochya chengi* sp. nov. **A** antenna, male **B** antenna, female **C** aedeagus, dorsal view **D** aedeagus, lateral view **E** aedeagus, ventral view **F** gonocoxae **G** abdominal ventrite VIII **H** spermatheca.

***Length*** 4.2–5.2 mm, width 2.3–3.0 mm. ***General color*** reddish brown (Fig. 4A–C); antennae blackish brown except two basal antennomeres reddish brown. ***Antennae*** (Fig. 5A) filiform in males, ratio of length of antennomeres I to XI 1.0 : 0.4 : 0.4 : 0.9 : 1.0 : 1.0 : 1.1 : 0.9 : 0.9 : 0.8 : 1.0; ratio of length to width from antennomere I to XI 4.7 : 1.9 : 2.2 : 4.5 : 5.0 : 5.3 : 5.3 : 4.8 : 4.6 : 4.1 : 5.5; a little slender in females, ratio of length of antennomeres I to XI (Fig. 5B) 1.0 : 0.4 : 0.4 : 1.0 : 1.0 : 1.0 : 1.0 : 0.9 : 0.8 : 0.7 : 0.9; ratio of length to width from antennomere I to XI 3.6 : 1.9 : 2.4 : 5.3 : 5.8 : 5.8 : 5.4 : 4.8 : 4.9 : 4.7 : 5.1. ***Pronotum*** 1.73–1.76 times wider than long; lateral margins slightly rounded and basally narrowed, basal margin slightly rounded, apical margin slightly concave; disc with dense coarse punctures, without lateral depressions. ***Elytra*** 1.33–1.42 times longer than wide; parallel sided; disc slightly convex, with dense, coarse punctures; apex truncate. ***Penis*** (Fig. 5C–E) wide, ca. 3.5 times longer than wide; lateral margins parallel from base to middle, then slightly narrowed towards apex, apex broadly rounded; tectum broad from apical 1/6 to middle, apex truncate; slightly and curved at apical 1/3 in lateral view; ventral surface with membranous area from apex to apical 1/3. Endophallic spiculae complex with median endophallic spiculae composed of seven pairs of hooked spiculae, and ventral endophallic spiculae composed of four pairs of hooked spiculae; with one pair of longitudinal rows of hair-like setae and one pair of longitudinal double rows of small stout setae near base. ***Gonocoxae*** (Fig. 5F) slender, tightly conjunct from apex to apical 1/3; each gonocoxa with eight setae from apical 1/6 to apex, subapically widened, apex truncate. ***Ventrite*** VIII (Fig. 5G) weakly sclerotized except apex, with several long setae at apex, and several long setae at sides, spiculum elongate. ***Spermathecal receptaculum*** (Fig. 5H) as slender as pump, apically tapering; pump slender and curved; sclerotized spermathecal duct extremely elongate, but base extremely wide, followed by very short slender tube, then followed with inflated areas. Bursal sclerites reduced.

#### Variation.

Most of specimens from Tahanshan have a distinct color pattern on elytra (Fig. 4D, E): with two pairs of transverse, wide black bands, running from lateral margins, abbreviated before suture, anterior pair near base, posterior pair at apical 1/3 an oblique; with one transverse, broad white band at middle. One specimen from Kenting has much narrower black bands on the elytra (Fig. 4F).

#### Diagnosis.

*Neochya chengi* sp. nov. is similar to *N. nitidissima* (Chûjô) in having wide elytra, truncate elytral apices and reduced lateral depressions on the pronotum (Figs 4, 9) (narrow elytra, rounded elytra apices and with lateral depression on the pronotum in others (Figs 2D–F, 7) but it differs from *N. nitidissima* in paralle sided elytra and having coarse punctures on pronotum and elytra (Fig. 4) (rounded elytra and reduced punctures on pronotum and fine punctures on elytra in *N. nitidissima* (Fig. 9)), and parallel sided elytra (rounded elytra in *N. nitidissima*). In addition, males of both species are separated from others with smooth margin of tectum of the penis (Figs 5C, 10C) (serrate margin of tectum (Figs 8C, 12C), but males of *N. chengi* differs from those of *N. nitidissima* with absence of small rounded process on lateral margin of the penis (Fig. 5C–E) (with small rounded process on lateral margin of the penis in *N. nitidissima* (Fig. 10C–E)

#### Food plants.

Celastraceae: *Celastrus hindsii* Benth (Fig. 1A), *C. kusanoi* Hayata.

#### Etymology.

It is named after Mr. Hsing-Tzung Cheng who was a member of the TCRT and an editor for a series of the books entitled “The Chrysomelidae of Taiwan”. The gender is feminine.

#### Distribution.

**Figure 6.**
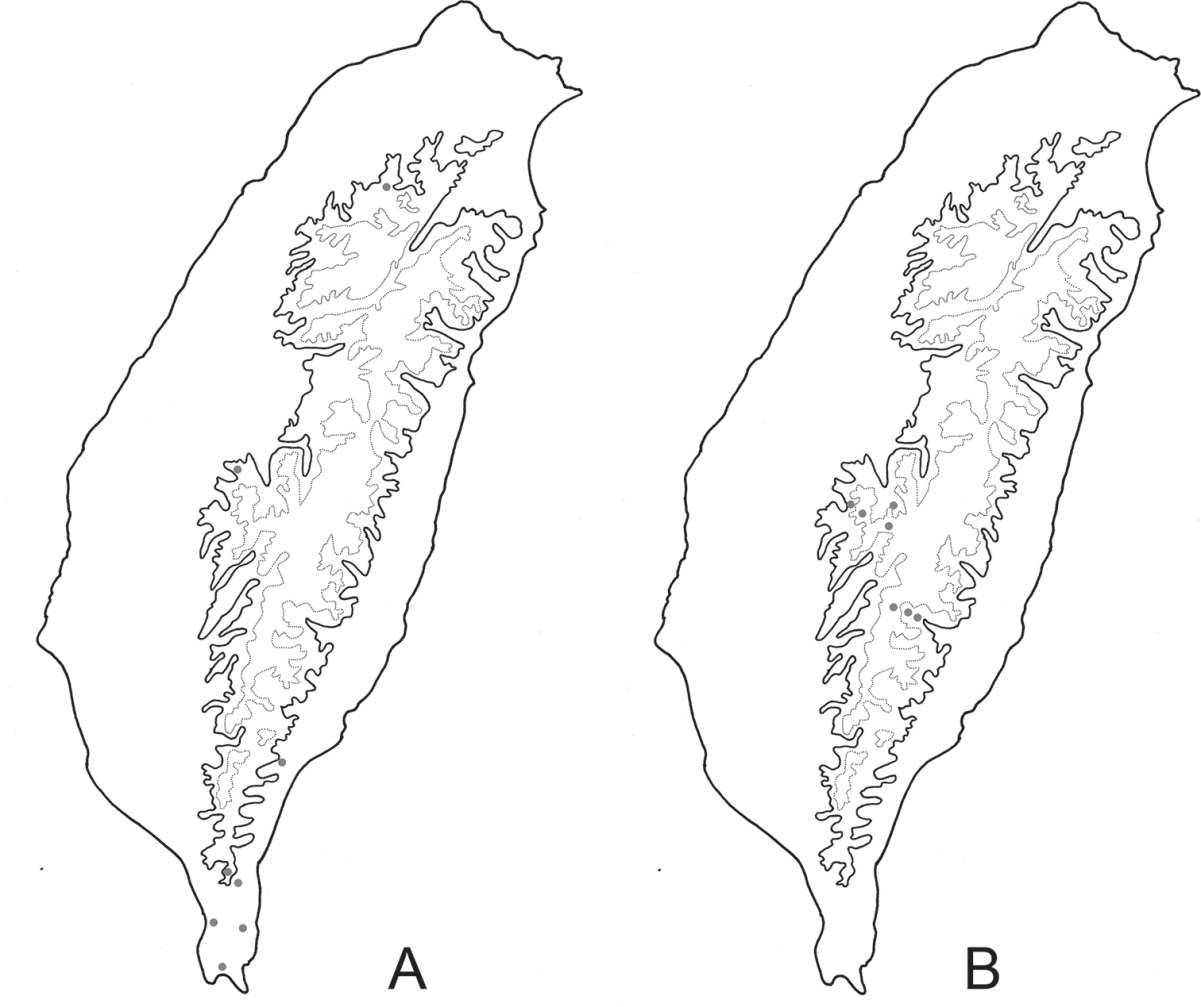
Distribution map of *Neochya* species, solid line: 1000 m, broken line: 2000 m **A***N. chengi* sp. nov. **B***N. hirashimai*.

Widespread but scattered in Taiwan (Fig. 6A).

### Neochya hirashimai

Taxon classificationAnimaliaColeopteraChrysomelidae

(Kimoto) comb. nov.

D6028757-1927-57D0-906C-95813B5ED5E7

Figures 7 , 8

Atrachya hirashimai Kimoto, 1969: 54; Kimoto 1989: 257.

#### Types.

***Holotype*** ♂ (KUEC): “(Taiwan) / Alishan, 2300m / Chiayi Hisen [p, w] // 9 [h].IV.1965 / Y. Hirashima [p, w] // Japan –U. S. / Co-op. Sci. / Programme [p, y] // Atrachya / hirashimai / Kimoto, n. sp. [h, w] // HOLOTYPE [p, r]. ***Paratypes***: 1♂ (KMNH), same data as holotype but with “PARATOPOTYPE [p, b]”; 1♂ (KMNH): “(Taiwan) / Fenchihu (奮起湖), 1400 m / Chiayi Hsien [p, w] // 12[h].IV.1965 / T. Shirôzu [p, w] // Japan –U. S. / Co-op. Sci. / Programme [p, y] // Atrachya / hirashimai / Kimoto, n. sp. [h, w] // PARATYPE [p, b]”.

#### Other material

**(*N* = 81). Chiayi**: 4♂♂, 4♀♀ (TARI), Alishan (阿里山), 12.V.2011, leg. C.-F. Lee; 2♂♂, 1♀ (TARI), Shihshan catchwater, 23.XI.2013, leg. W.-C. Liao; 3♂♂, 3♀♀ (TARI), same locality, 21.I.2017, leg. W.-C. Liao; **Nantou**: 1♀ (TARI), Tatachia (塔塔加), 15–22.IV.2007, leg. C.-S. Tung; 1♀ (TARI), same locality, 9.VI.2009, leg. C.-F. Lee; 9♂♂, 3♀♀ (TARI), same locality, 30.X.2009, leg. C.-F. Lee; 2♂♂ (TARI), same locality, 29–30.XII.2009, leg. H. Lee, M.-H. Tsou, H. Lee; 8♂♂, 6♀♀ (TARI), same locality, 27.IV.2010, leg. C.-F. Lee; 5♂♂, 6♀♀ (TARI), same locality, 16–17.V.2010, leg. C.-F. Lee & M.-H. Tsou; 2♂♂, 4♀♀ (TARI), same locality, 8–9.V.2011, leg. C.-F. Lee & M-H. Tsou; 1♀ (TARI), Tungpu (東埔), 8.V.2015, leg. J.-C. Chen; **Taitung**: 2♂♂, 1♀ (TARI), Hsiangyang (向陽), 5.IV.2012, leg. J.-C. Chen; 1♀ (TARI), same locality, 3.VII.2014, leg. J.-C. Chen; 4♂♂, 4♀♀ (TARI), Liyuan (栗園), 23.V.2011, leg. C.-F. Lee; 1♂, 3♀♀ (TARI), Motien (摩天), 19.VI.2011, leg. C.-F. Lee.

#### Redescription.

**Figure 7.**
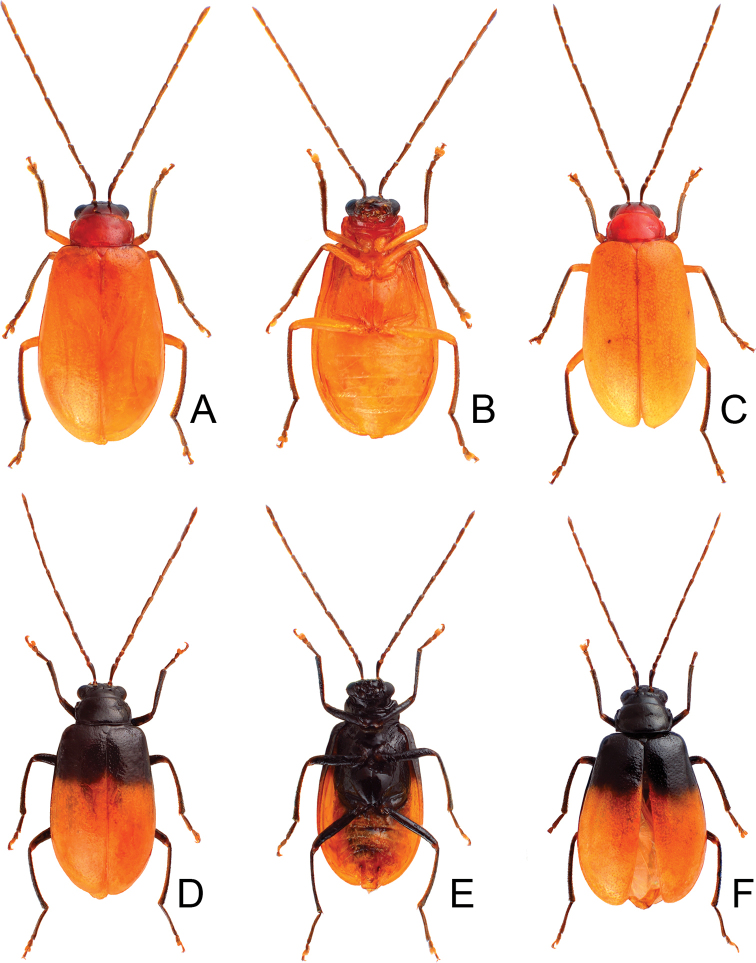
Habitus of and *Neochya hirashimai***A** male, from Tatachia (塔塔加), dorsal view **B** same, ventral view **C** female, from Tatachia (塔塔加), dorsal view **D** male, from Liyuan (栗園), dorsal view **E** same, ventral view **F** female, from Liyuan (栗園), dorsal view.

**Figure 8.**
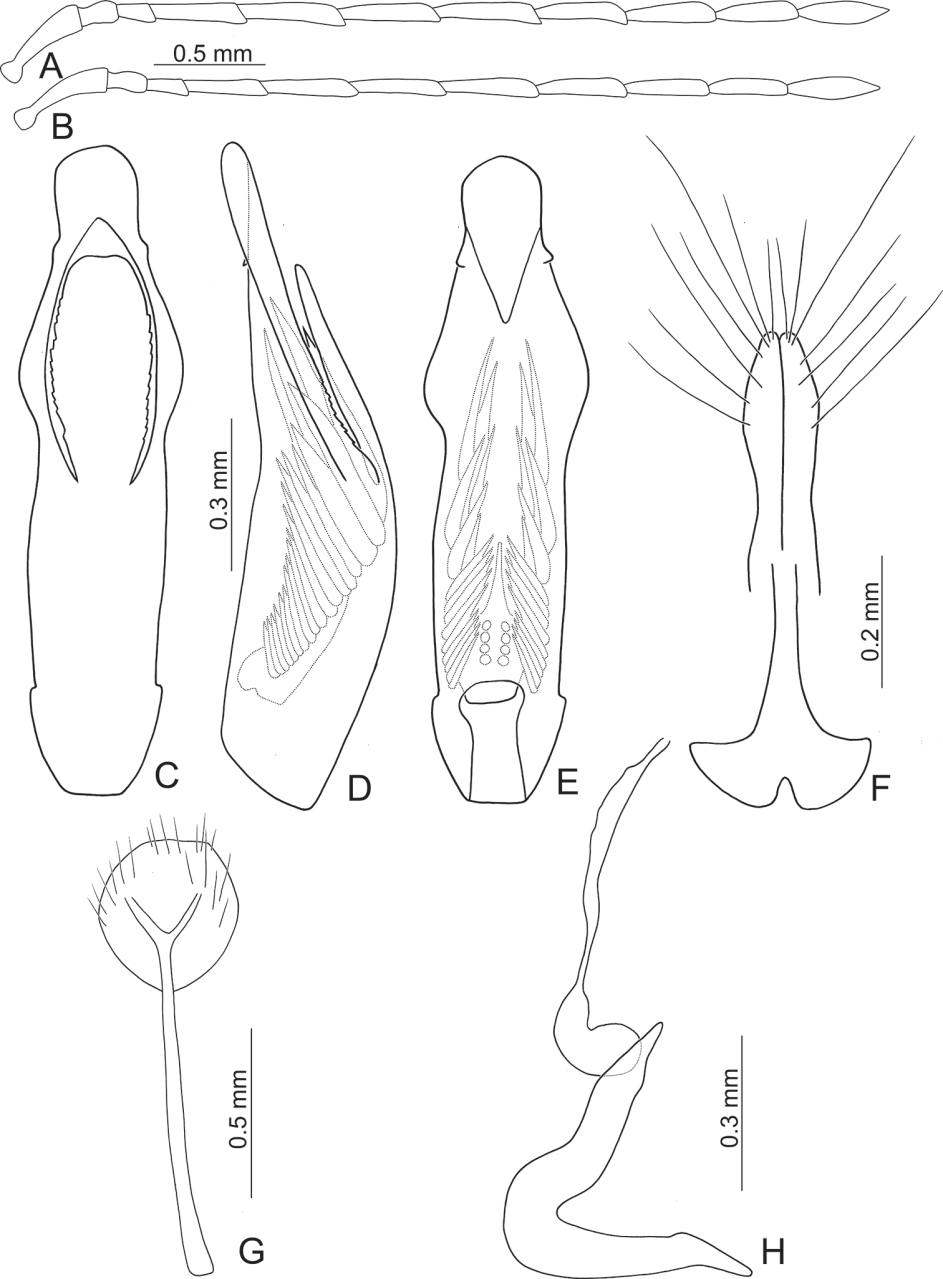
Diagnostic characters of *Neochya hirashimai***A** antenna, male **B** antenna, female **C** aedeagus, dorsal view **D** aedeagus, lateral view **E** aedeagus, ventral view **F** gonocoxae **G** abdominal ventrite VIII **H** spermatheca.

***Length*** 5.1–6.2 mm, width 2.4–3.1 mm. ***General colo***r yellowish brown (Fig. 7A–C); prothorax and head reddish brown, but anterior portion of head darker; antennae, tibiae, and tarsi black. ***Antennae*** (Fig. 8A) filiform in males, ratio of length of antennomeres I to XI 1.0 : 0.4 : 0.4 : 1.0 : 1.1 : 1.1 : 1.1 : 1.0 : 1.0 : 0.9 : 1.0; ratio of length to width from antennomere I to XI 3.6 : 1.7 : 1.8 : 4.5 : 5.1 : 5.3 : 5.3 : 4.7 : 4.5 : 4.1 : 4.4; slender in females, ratio of length of antennomeres I to XI (Fig. 8B) 1.0 : 0.4 : 0.4 : 0.8 : 1.0 : 0.9 : 1.0 : 0.8 : 0.8 : 0.8 : 0.9; ratio of length to width from antennomere I to XI 4.2 : 2.0 : 2.4 : 4.7 : 5.7 : 5.5 : 5.7 : 5.1 : 5.2 : 5.2 : 3.9. ***Pronotum*** 1.92–2.00 times wider than long; lateral margins straight and basally narrowed, basal margin slightly rounded, apical margin slightly concave; disc with dense coarse punctures, with lateral depressions. ***Elytra*** 1.57–1.68 times longer than wide; lateral margins widest at apical 1/3; disc slightly convex, with dense, coarse punctures; apex rounded. ***Penis*** (Fig. 8C–E) wide, ca. 4.0 times longer than wide; lateral margins parallel from base to middle, then strongly broadened, widest at apical 1/3; apex broadly rounded; tectum elongate from apical 1/6 to middle, lateral margins slightly rounded and toothed, apex truncate but medially depressed; weakly curved in middle in lateral view; ventral surface with membranous area from apex to apical 1/4; with one small rounded process on lateral margin at apical 1/6. Endophallic spiculae complex, with five or six pairs of hooked spiculae; with one pair of longitudinal rows of hair-like setae and small rounded sclerites near base. ***Gonocoxae*** (Fig. 8F) slender, tightly conjunct from apex to middle; each gonocoxa with eight setae from apical 1/6 to apex, subapically widened, apex narrowly rounded, base shallowly bifurcate. ***Ventrite*** VIII (Fig. 8G) weakly sclerotized except apex, with several long setae at apex, and several long setae at sides, spiculum elongate. ***Spermathecal receptaculum*** (Fig. 8H) as slender as pump, apically tapering; pump slender and curved; sclerotized spermathecal duct extremely elongate, but base extremely wide, followed by slender tube, then with inflated areas. Bursal sclerites reduced.

#### Variation.

A distinct color pattern occurs in beetles from the east part of South Cross-Island Highway (南橫公路): general color black; but apical 3/4 of elytra and abdomen yellowish brown (Fig. 7C–E).

#### Diagnosis.

**Figure 9.**
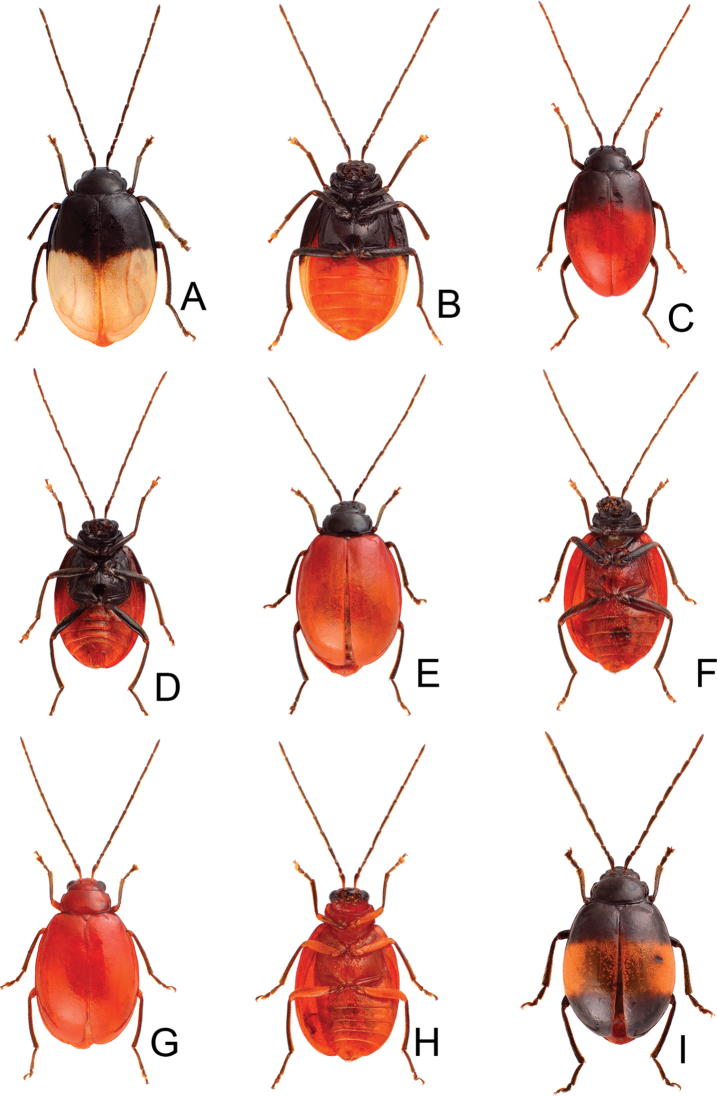
Habitus of *Neochya nitidissima***A** form A, female, from Alishan (阿里山), dorsal view **B** same, ventral view **C** form B, male, from Kuanwu (觀霧), dorsal view **D** same, ventral view **E** form C, female, from Pilu (碧綠), dorsal view **F** same, ventral view **G** form D, female, from Taikang trial (泰崗林道), dorsal view **H** same, ventral view **I** form E, male, from Peitawushan (北大武山), dorsal view.

**Figure 10.**
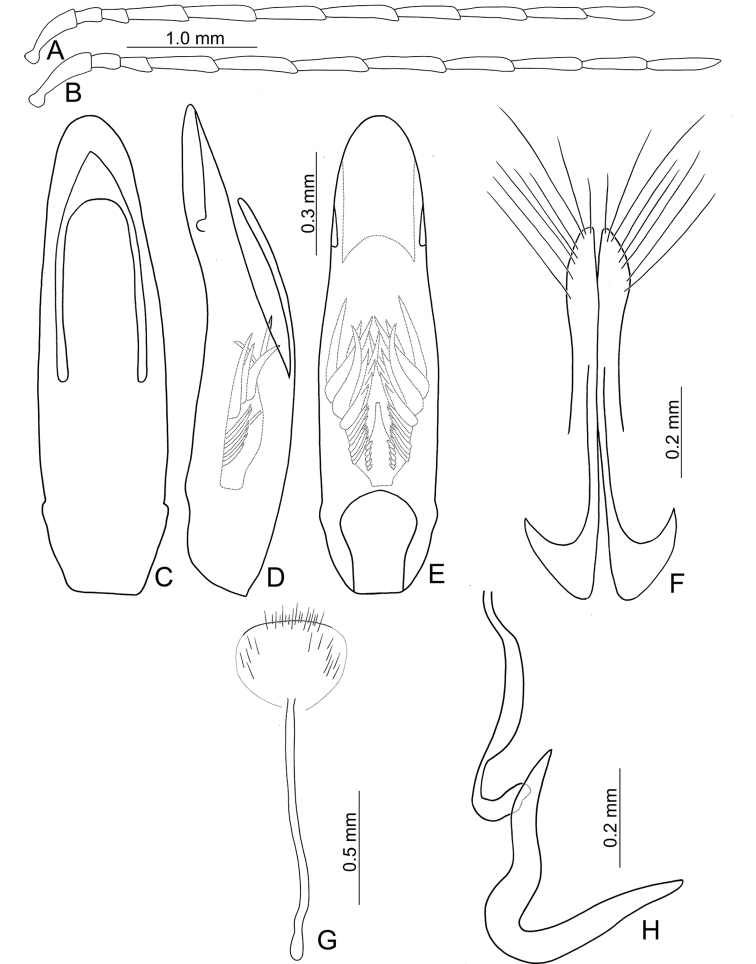
Diagnostic characters of *Neochya nitidissima***A** antenna, male **B** antenna, female **C** aedeagus, dorsal view **D** aedeagus, lateral view **E** aedeagus, ventral view **F** gonocoxae **G** abdominal ventrite VIII **H** spermatheca.

*Neochya hirashimai* (Kimoto) is similar to *N. tsoui* sp. nov. in having slender elytra and lateral depressions on the pronotum (Figs 2D–F, 7) (wide elytra and lacking lateral depressions on the prnotum in others (Figs 4, 9), but differs from *N. tsoui* sp. nov. in having reddish brown pronotum and yellowish brown elytra, or black pronotum, black basal half and yellowish brown apical half of elytra (Fig. 7) (reddish brown pronotum and elytra in *N. tsoui* sp. nov. (Fig. 2D–F)). In addition, males of both species are separated from others with serrate margin of tectum of the penis (Figs 8C, 12C) (smooth margin of tectum (Figs 5C, 10C), but males of *N. hirashimai* differs from those of *N. tsoui* with the penis widest at middle (Fig. 8C, E) (penis widest at apical 2/5 in *N. nitidissima* (Fig. 12C, E).

#### Food plants.

Celastraceae: *Celastrus kusanoi* Hayata (Fig. 1B, C), *C. punctatus* Thunb.

#### Distribution.

Restricted to several places in central Taiwan. Two color patterns are separated in the eastern and western parts of the range (Fig. 6B).

### Neochya nitidissima

Taxon classificationAnimaliaColeopteraChrysomelidae

(Chûjô, 1935) comb. nov.

FE167CDA-4D25-51EC-8C21-D81383383731

Figures 9 , 10

Luperodes nitidissimus Chûjô, 1935: 161; Chûjô 1962: 228.
Atrachya nitidissimus : Kimoto, 1966: 30 (additional records); Kimoto 1969: 55(additional records).
Luperodes bicoloripennis Chûjô, 1938: 138; Chûjô 1962: 227 (redescription). syn. nov.
Atrachya bicoloripennis : Kimoto, 1969: 55: (additional records); Kimoto 1986: 58 (additional records); Kimoto 1987: 190 (additional records).
Atrachya bicolor [sic!]: Kimoto, 1989: 257 (additional records).
Luperodes saramao Chûjô, 1962: 230. syn. nov.
Atrachya saramao : Kimoto, 1989: 257 (additional records).

#### Types.

*Luperodes nitidissimus*. ***Holotype*** ♀ (SDEI): “Kankau (Koshun = 恆春) / Formosa / H. Sauter V. 1912 [p, w] // Luperodes / nitidissimus / Chûjô [h] / DET. M. CHUJO [b, w] // Holotype [p, w, red letters]”. ***Paratypes*.** 1♀ (TARI): “Formosa / Koshun, 1918 / IV 25–V 28 / J. Sonan, [p, w] // Allotype [h, w, red letters] // Luperodes / nitidissimus / Chûjô [h] / DET. M. CHUJO [b, w] // 2966 [p, w]”; 1♀ (TARI): “Formosa / Koshun, 1918 / IV 25–V 28 / J. Sonan, [p, w] // Paratype [h, w, red letters] // Luperodes / nitidissimus / Chûjô [h] / DET. M. CHUJO [b, w] // 2967 [p, w]”.

*Luperodes bicoloripennis*. ***Lectotype*** ♂ (TARI, here designated): “Arisan (阿里山) / FORMOSA/ 24–25.V.1933 / Col. M. CHUJO [p, w] // CO / Type [p, w, circular label with yellow letters] // Luperodes / bicoloripennis / Chûjô [h] / DET. M. CHUJO [b, w] // 1383 [p, w]”. ***Paralectotypes***: 1♀ (TARI), same data as holotype but with “1382”; 1♂ (TARI): “Arisan [h] / FORMOSA [p] / 22.X.1931 [h] / COL. M. CHUJO [p, w] // CO / Type [p, w, circular label with yellow letters] // Luperodes / bicoloripennis / Chûjô [h] / DET. M. CHUJO [b, w] // 1381 [p, w]”; 1♀ (TARI), same but with “1373”; 1♂ (SDEI): “Arisan / FORMOSA / 24–25.V.1933 / COL. M. CHUJO [p, w] // Luperodes / bicoloripennis / Chûjô [h] / DET. M. CHUJO [b, w] // Syntypus [p, r]” 1♀ (SDEI), same but with “25.V.1933 [p]”; 1 (TARI, sex undetermined, abdomen lost): “ARISAN / XII.1915 [h] / Coll. M. Maki [p, w] // CO / Type [p, w, circular label with yellow letters] // Luperodes / bicoloripennis / Chûjô [h] / DET. M. CHUJO [b, w] // 1493 [p, w]”; 1♂ (TARI): “Formosa / Musha (霧社), 1919 / V 18–VI 15. / T. Okuni, [p, w] // CO / Type [p, w, circular label with yellow letters] // Luperodes / bicoloripennis / Chûjô [h] / DET. M. CHUJO [b, w] // 2587 [p, w]”; 1♀ (TARI), same but with “1492”. One specimen (♀, TARI) bearing type label: “Jujiro (十字路, in Chiayi) / 26-IV 1931 / Col. T. Shiraki [p, w] // CO / Type [p, w, circular label with yellow letters] // Luperodes / bicoloripennis / Chûjô [h] / DET. M. CHUJO [b, w] // 1494 [p, w]”. It cannot be included in the type series since the data did not appear in the original description (Chûjô 1938).

*Luperodes saramao*. ***Holotype*** ♂ (KUEC): “13 VIII 1936 / Mururoahu (給里洛山 =見晴山) / --- Kussya (庫霞 = 大同) / TAIHEIZAN (太平山) [p, pink label] // Luperodes / saramao / Chûjô [h, w] // Holotype [h, r]”.

#### Other material.

**Form A (*N* = 84). Chiayi**: 1♂, 1♀ (TARI), Alishan (阿里山), 12.V.2011, leg. C.-F. Lee; 1♀ (TARI), same locality, 29.V.2016, leg. B.-X. Guo; 5♂♂, 1♀ (TARI), Shihshan channel (石山引水道), 23.XI.2013, leg. W.-C. Liao; 1♂, 2♀♀ (TARI), same locality, 21.I.2017, leg. W.-C. Liao; 8♂♂, 8♀♀ (TARI), Tzuchung (自忠), 21.IX.2009, leg. C.-F. Lee; **Kaohsiung**: 1♂ (TARI), Tienchih (天池), 17.V.2015, leg. B.-X. Guo; **Nantou**: 2♂♂ (TARI), Fenghuangshan (鳳凰山), 10.V.2010, leg. Y.-T. Wang; 2♂♂ (TARI), Hsitou (溪頭), 6.V.2009, leg. C.-F. Lee; 1♂ (NMNS), Shanlinchi (杉林溪), 10.V.1990, leg. C.-C. Chiang; 2♀♀ (NMNS), same locality, 19.V.1991, leg. C.-C. Chiang; 2♂♂, 3♀♀ (TARI), Tatachia (塔塔加), 9.VI.2009, leg. C.-F. Lee; 1♂, 1♀ (TARI), same locality, 21.VII.2009, leg. S.-F. Yu; 1♂, 1♀ (TARI), same locality, 21.IX.2009, leg. C.-F. Lee; 2♂♂ (TARI), same locality, 29.X.2009, leg. H. Lee; 1♂, 1♀ (TARI), same locality, 30.X.2009, leg. C.-F. Lee; 1♂, 2♀♀ (TARI), same locality, 17.XI.2009, leg. C.-F. Lee; 2♀♀ (TARI), same locality, 29.XII.2009, leg. M.-H. Tsou; 4♂♂, 4♀♀ (TARI), same locality, 17.XI.2009, leg. C.-F. Lee; 3♂♂ (TARI), same locality, 27.IV.2010, leg. C.-F. Lee; 1♂, 1♀ (TARI), same locality, 17.V.2010, leg. M.-H. Tsou; 2♂♂ (TARI), same locality, 13.VII.2014, leg. W.-C. Liao; **Taichung**: 1♂ (TARI), Anmashan (鞍馬山), 6–9.VII.1979, leg. L.-Y. Chou; 1♂ (TARI), same locality, 18.X.2008, leg. H. Lee; 2♂♂ (TARI), same locality, 15.X.2009, leg. J.-C. Chen; 1♀ (TARI), same locality, 7.VI.2010, leg. C.-F. Lee.

**Form B (*N* = 255). Hsinchu**: 1♀ (TARI), Kuanwu (觀霧), 30.IV.2009, leg. Y.-F. Hsu; 1♀ (TARI), same locality, 19.VIII.2009, leg. Y.-F. Hsu; 1♂ (TARI), same locality, 5.XI.2009, leg. H. Lee; 27♂♂, 7♀♀ (TARI), same locality, 30.IV.–1.V.2010, leg. C.-F. Lee & M.-H. Tsou; 1♂ (TARI), 3.VI.2011, leg. S.-F. Yu; 1♂ (TARI), Talulintao (大鹿林道), 17.II.2008, leg. M.-H. Tsou; 1♀ (TARI), same locality, 25.III.2009, leg. Y.-L. Lin; 2♂♂, 1♀ (TARI), same locality, 23.VI.2016, leg. Y.-L. Lin; **Hualien**: 2♂♂, 2♀♀ (TARI), Tayuling (大禹嶺), 2.VI.2016, leg. Y.-T. Chung; **Miaoli**: 1♂, 1♀ (TARI), Hsuehchien (雪見), 7.VI.2013, leg. W.-B. Yeh; 4♂♂ (TARI), Leshan (樂山), 14.VIII.2010, leg. H.-J. Chen; **Nantou**: 5♂♂, 3♀♀ (TARI), Huakung (華岡), 12.–13.IX.2010, leg. C.-F. Lee; 1♂ (TARI), same locality, 17.VI.2016, leg. J.-C. Chen; 1♂ (TARI), Lienhuachi (連華池), 23.–26.V.1980, leg. K.-S. Lin & B.-H. Chen; 3♂♂, 2♀♀ (TARI), Meifeng (梅峰), 10.V.1979, leg. K.-C. Chou; 2♂♂, 4♀♀ (TARI), same locality, 20.–22.VI.1979, leg. K.-S. Lin & B.-H. Chen; 1♂ (TARI), same locality, 23.V.–3.VI.1979; 1♀ (TARI), same locality, 18.VII.1979, leg. K.-C. Chou; 1♀ (TARI), same locality, 2.–12.X.1979; 1♂ (TARI), same locality, 24.X.1979, leg. K.-C. Chou; 1♂, 1♀ (TARI), same locality, 2.–4.VI.1980, leg. L.-Y. Chou & C.-C. Chen; 1♂, 1♀ (TARI), same locality, 5.–8.VI.1980, leg. C.-C. Chen; 2♂♂, 4♀♀ (TARI), same locality, 8.VI.1980, leg. K.-S. Lin & B.-H. Chen; 1♀ (TARI), same locality, 9.–16.VI.1980, leg. K.-S. Lin & B.-H. Chen; 6♂♂, 3♀♀ (TARI), same locality, 22.V.1982, leg. L.-Y. Chou; 3♂♂, 2♀♀ (TARI), same locality, 4.–7.X.1982, leg. K.-C. Chou; 1♂, 1♀ (TARI), same locality, 19–21.IV.1983, leg. K.-C. Chou & S.-P. Huang; 1♀ (TARI), same locality, 30.VII.1983, leg. L.-Y. Chou; 1♀ (TARI), same locality, 8.-11.V.1984, leg. K.-C. Chou & C.-C. Pan; 1♂ (TARI), same locality, 23.VII.1984, leg. K.-S. Lin; 1♀ (NMNS), same locality, 3.-15.X.1990, leg. C. K. Starr; 2♂♂ (NMNS), same locality, 27.II.1992, leg. Y.-C. Shiau; 1♀ (NMNS), same locality, 6.VIII.–11.IX.2001, leg. C.-S. Lin & W.-T. Yang; 5♀♀ (NMNS), same locality, 15.IV.–7.V.2003, leg. C.-S. Lin & W.-T. Yang; 2♀♀ (NMNS), same locality, 7.V.–11.VI.2003, leg. C.-S. Lin & W.-T. Yang; 1♂ (NMNS), same locality, 4.XI.–15.XII.2003, leg. C.-S. Lin & W.-T. Wang; 1♀ (NMNS), same locality, 6.IV.–11.V.2004, leg. C.-S. Lin & W.-T. Yang; 1♀ (NMNS), same locality, 5.X.–16.XI.2004, leg. C.-S. Lin & W.-T. Yang; 1♀ (NMNS), same locality, 12.IV.3.V.2005, leg. C.-S. Lin & W.-T. Yang; 1♀ (NMNS), same locality, 20.II.2009, leg. M.-L. Chan; 1♀ (TARI), same locality, 18.V.2009, leg. M.-H. Tsou; 2♀♀ (TARI), same locality, 15.IX.2009, leg. H. Lee; 2♀♀ (TARI), same locality, 15.IX.2009, leg. S.-F. Yu; 1♀ (TARI), same locality, 17.VI.2010, leg. C.-F. Lee; 2♂♂, 3♀♀ (TARI), same locality, 20.IV.2011, leg. C.-F. Lee; 1♂ (TARI), same locality, 30.V.2011, leg. M.-H. Tsou; 25♂♂, 18♀♀ (TARI), Peitungyanshan (北東眼山), 16.IX.2013, leg. F.-S. Huang; 2♀♀ (TARI), same locality, 28.III.2014, leg. F.-S. Huang; 3♂♂ (TARI), same locality, 14.IV.2014, leg. C.-F. Lee; 1♀ (NMNS), Piluchi (碧綠溪), 4.XII.1991, leg. Y.-C. Shiau; 1♂ (NMNS), same locality, 15.–31.III.1998, leg. M.-M. Yang; 3♂♂, 3♀♀ (TARI), Sungkang (松崗), 15.-17.VIII.1984, leg. K.-C. Chou; 1♂ (TARI), same locality, 18.IV.2015, leg. B.-X. Guo; 1♂, 1♀ (TARI), same locality, 10.IV.2016, leg. Y.-T. Chung; 1♂ (TARI), same locality, 2.VI.2016, leg. B.-X. Guo; 1♂, 3♀♀ (TARI), Tsuifeng (翠峰), IV.1984, leg. K.-S. Lin & K.-C. Chou; 1♂ (NMNS), Tunyuan (屯原), 29.IV.1992, leg. W.-T. Yang; 4♂♂, 1♀ (TARI), same locality, 18.X.2011, leg. J.-C. Chen; 1♂ (TARI), same locality, 30.IV.2017, leg. Y.-F. Hsu; 1♂ (NMNS), Yuanfeng (鳶峰), 5.VII.–2.VIII.2005, leg. C.-S. Lin & W.-T. Yang; **Taichung**: 9♂♂, 9♀♀ (TARI), Anmashan (鞍馬山), 6.–9.VII.1979, leg. L.-Y. Chou; 1♀ (NMNS), same locality, 1.V.1990, leg. C.-C. Chiang; 2♂♂ (NMNS), same locality, 3.V.1992, leg. C.-Y. Li; 1♂, 1♀ (NMNS), 22.IV.1998, leg. M.-M. Yang & H.-T. Chan; 1♀ (TARI), same locality, 16.VII.2007, leg. M.-H. Tsou; 1♀ (TARI), same locality, 7.VI.2010, leg. C.-F. Lee; 1♂ (TARI), same locality, 23.VII.2011, leg. J.-C. Chen; 9♂♂, 10♀♀ (TARI), Fushoushan (福壽山), 3.V.2016, leg. J.-C. Chen; 1♀ (NMNS), Nanhushi (南湖溪), 10.–11.VI.1988, leg. K.-W. Huang; 1♂ (TARI), Pilu (畢祿), 22.IV.2015, leg. C.-F. Lee; 1♀ (TARI), Wuleng (武陵), 27.–29.VI.1979, leg. K.-S. Lin & L.-Y. Chou; 1♂ (TARI), same locality, 6.IV.2014, leg. J.-C. Chen; 1♂ (TARI), same locality, 15.VIII.2014, leg. M.-H. Tsou.

**Form C (*N* = 85). Hsinchu**: 1♂ (TARI), Sumakusu (司馬庫斯), 26.IX.2009, leg. H.-J. Chen; **Hualien**: 1♀ (TARI), Kuanyuan (關原), 7.V.2006, leg. Y.-F. Hsu; 1♀ (TARI), same locality, 2.VII.2008, leg. M.-H. Tsou; 1♂, 2♀♀ (TARI), same locality, 2.VI.2016, leg. Y.-T. Chung & B.-X. Guo; 1♂ (NMNS), Kuanyun (觀雲), 13.V.2005, leg. J.-H. Chen; 1♂ (TARI), same locality, 19.VI.2010, leg. W.-P. Chan; 1♀ (TARI), Pilu (碧綠), 9.VII.2009, leg. C.-F. Lee; 1♀ (TARI), same locality, 10.IV.2014, leg. C.-F. Lee; 1♂ (TARI), same locality, 13.VI.2014, leg. C.-F. Lee; 1♀ (TARI), same locality, 23.IV.2015, leg. C.-F. Lee; 1♂ (TARI), same locality, 14.V.2015, leg. J.-C. Chen; 1♂, 1♀ (TARI), same locality, 7.VII.2015, leg. C.-F. Lee; 1♀ (TARI), same locality, 22.VII.2015, leg. U. Ong; 1♂, 2♀♀ (TARI), Sungyuan (松苑), 19.V.2018, leg. H.-F. Lu; 1♂ (TARI), Tayuling (大禹嶺), 6.–9.IX.1983, leg. L.-Y. Chou & K.-C. Chou; **Kaohsiung**: 2♀♀ (TARI), Chungchihkuan (中之關), 16.IV.2012, leg. L.-P. Hsu; 1♀ (TARI), same locality, 12.X.-6.XII.2012, leg. L.-P. Hsu; 1♂, 1♀ (TARI), same locality, 31.VII.2015, leg. C.-F. Lee; 1♂ (TARI), Tengchih (藤枝), 2.–5.VI.2008, leg. C.-F. Lee; 1♀ (TARI), same locality, 7.–10.XI.2008, leg. C.-T. Yao; 1♀ (TARI), same locality, 6.II.2009, leg. S.-F. Yu; 1♀ (TARI), same locality, 23.III.2009, leg. H. Lee; 1♀ (TARI), 26.V.2009, leg. C.-F. Lee; 1♀ (TARI), same locality, 6.VIII.2013, leg. B.-X. Guo; 1♀ (TARI), same locality, 8.III.2014, leg. W.-C. Liao; 3♂♂, 3♀♀ (NMNS), Tona (多納), 28.IV.1998, leg. M.-L. Chan; 1♀ (TARI), same locality, 2.VIII.2017, leg. B.-X. Guo; **Miaoli**: 1♂ (TARI), Hsuehchien (雪見), 12.III.2013, leg. W.-B. Yeh; 2♂♂ (TARI), same locality, 7.VI.2013, leg. W.-B. Yeh; 2♀♀ (NMNS), Taian (泰安), 19.XII.1989, leg. K.-W. Huang; **Nantou**: 1♂, Tunyuan (屯原), 12.VII.2014, leg. J.-C. Chen; **Pingtung**: 1♂ (TARI), Peitawushan (北大武山), 22.IX.2012, leg. J.-C. Chen; 1♂ (TARI), same locality, 25.VI.2018, leg. Y.-T. Chung; 1♀ (TARI), Tahanshan (大漢山), 20.VII.2007, leg. C.-F. Lee; 3♂♂, 8♀♀ (TARI), same locality, 6.II.2008, leg. M.-H. Tsou & S.-F. Yu; 3♂♂ (TARI), same locality, 3.III.2008, leg. C.-F. Lee; 1♀ (TARI), same locality, 4.VII.2008, leg. M.-H. Tsou; 3♂♂, 1♀ (TARI), same locality, 22.I.2009, leg. S.-F. Yu; 1♀ (TARI), same locality, 24.I.2009, leg. M.-H. Tsou; 1♂, 2♀♀ (TARI), same locality, 5.IV.2009, leg. C.-F. Lee; 1♀ (TARI), same locality, 26.XI.2009, leg. J.-C. Chen; 1♀ (TARI), 15.II.2010, leg. M.-H. Tsou; 1♂ (TARI), same locality, 14.IV.2011, leg. J.-C. Chen; 1♀ (TARI), same locality, 14.XII.2011, leg. J.-C. Chen; 1♀ (TARI), same locality, 19.XI.2012, leg. J.-C. Chen; 1♀ (TARI), same locality, 26.III.2013, leg. C.-F. Lee; 1♀ (TARI), same locality, 3.IV.2013, leg. Y.-T. Chung; 3♂♂ (TARI), same locality, 17.III.2014, leg. Y.-T. Chung & J.-C. Chen; 1♂ (TARI), same locality, 2.III.2015, leg. Y.-T. Chung; 1♀ (TARI), same locality, 24.III.2017, leg. Y.-T. Chung; **Taichung**: 1♂ (TARI), Pilu (畢祿), 18.VI.2010, leg. C.-F. Lee; 1♂ (TARI), same locality, 2.VI.2016, leg. J.-C. Chen.

**Form D (*N* = 44). Chiayi**: 4♂♂, 1♀ (TARI), Laichitashan (來吉塔山), 19.III.2009, leg. H. Lee; **Hsinchu**: 1♂, 1♀ (TARI), Leetungshan (李棟山), 15.III.2009, leg. S.-F. Yu; 4♂♂, 1♀ (TARI), same locality, 27.X.2009, leg. S.-F. Yu; 1♀ (TARI), Mamei (馬美), 4.V.2008, leg. S.-F. Yu; 1♀ (TARI), Taikang trial (泰崗林道), 8.IX.2013, leg. Y.-L. Lin; **Ilan**: 1♀ (TARI), Suyuan (思源), 15.IV.2009, leg. M.-H. Tsou; 1♀ (TARI), same locality, 9.VI.2009, leg. S.-F. Yu; 2♀♀ (TARI), same locality, 12.IX.2010, leg. M.-H. Tsou; 1♂, 1♀ (TARI), same locality, 11.VIII.2014, leg. H. Lee & J.-C. Chen; 1♂ (TARI), same locality, 30.VII.2015, leg. H. Lee; 1♀ (TARI), Taipingshan (太平山), 13.VI.2007, leg. Y.-C. Chang; **Pingtung**: 1♀ (TARI), Kenting (墾丁), 23.VIII.2016, leg. Y.-T. Chung; **Tainan**: 1♂ (TARI), Kantoushan (崁頭山), 17.VI.2012, leg. W.-C. Liao; 1♂, 1♀ (TARI), Meiling (梅嶺), 28.XII.2008, leg. U. Ong; **Taipei**: 1♂, 1♀ (TARI), Fushan (福山), 19.VI.2007, leg. M.-H. Tsou; 1♂ (TARI), Houtung (侯硐), 25.V.2009, leg. J.-C. Chen; **Taoyuan**: 1♂ (TARI), Lalashan (拉拉山), 7.VIII.2008, leg. H.-J. Chen; 1♂, 1♀ (TARI), same locality, 30.X.2008, leg. S.-F. Yu; 2♂♂, 2♀♀ (TARI), same locality, 8.III.2009, leg. S.-F. Yu; 1♂, 1♀ (TARI), same locality, 2.IV.2009, leg. C.-F. Lee & H.-J. Chen; 4♂♂, 1♀ (TARI), same locality, 14.V.2009, leg. C.-F. Lee; **Yunlin**: 1♀ (TARI), Shihpishan (石壁山), 26.IV.2015, leg. W.-C. Liao.

**Form E (*N* = 1). Pingtung**: 1♂ (TARI), Peitawushan (北大武山), 25.VI.2018, leg. Y.-T. Chung.

#### Redescription.

***Length*** 5.6–7.0 mm, width 2.9–3.8 mm. ***General color*** extremely variable, with five distinct color patterns (see variation). ***Antennae*** (Fig. 10A) filiform in males, ratio of length of antennomeres I to XI 1.0 : 0.4 : 0.4 : 1.0 : 1.1 : 1.1 : 1.1 : 1.0 : 1.1 : 0.9 : 1.0; ratio of length to width from antennomere I to XI 3.6 : 1.6 : 1.8 : 4.1 : 5.0 : 5.3 : 5.9 : 5.1 : 5.9 : 5.2 : 5.4; similar in females, ratio of length of antennomeres I to XI (Fig. 10B) 1.0 : 0.4 : 0.4 : 1.0 : 1.1 : 1.0 : 1.1 : 1.0 : 1.0 : 0.9 : 1.0; ratio of length to width from antennomere I to XI 3.6 : 1.8 : 2.1 : 4.5 : 5.3 : 5.2 : 5.4 : 5.5 : 5.5 : 5.0 : 5.8. ***Pronotum*** 1.90–1.94 times wider than long; lateral margins slightly rounded, basal margin slightly rounded, apical margin slightly concave; disc with dense minute punctures, but without lateral depressions. ***Elytra*** 1.36–1.40 times longer than wide; lateral margins rounded, widest at middle; disc moderately convex, with dense, minute punctures; apex truncate. ***Penis*** (Fig. 10C–E) wide, ca. 3.8 times longer than wide; lateral margins rounded, widest at basal 1/3; apex broadly rounded; tectum elongate from apical 1/6 to middle, parallel-sided, apex broadly rounded; slightly and apically curved in lateral view; ventral surface with membranous area from apex to apical 1/4; with one small rounded process on lateral margin at apical 1/4. Endophallic spiculae complex with six or seven pairs of hooked spiculae (visible in dorsal view), one additional pair of hooked spiculae near middle with four or five ventral branches; with one pair of longitudinal rows of hair-like setae and small rounded sclerites near base. ***Gonocoxae*** (Fig. 10F) slender, tightly conjunct from apical 1/6 to middle; each gonocoxa with eight setae from apical 1/6 to apex, subapically widened, apex narrowly rounded, base deeply bifurcate. ***Ventrite*** VIII (Fig. 10G) weakly sclerotized except apex, with several short and long setae at apex, and several long setae at sides, spiculum elongate. ***Spermathecal receptaculum*** (Fig. 10H) as slender as pump, apically tapering; pump slender and curved; sclerotized spermathecal duct extremely elongate, but base wide, followed by slender tube, then with inflated areas. Bursal sclerites reduced.

#### Variation.

Color pattern divided into four forms. Form A (Fig. 9A, B) (described as one form of *Atrachya bicoloripennis*): general color black; but apical 2/3 white, abdomen reddish brown. Form B (Fig. 9C, D): similar to form A, but white area replaced with red (described as another form of *A. bicoloripennis*). Form C (Fig. 9E, F) (described as *A. saramao*): similar to form B, but elytra entirely reddish brown, meso-and metathoracic ventrites reddish brown; some individuals have paler femora and antennae. Form D (Fig. 9G, H) (described as typical form of *A. nitidissima*): body color reddish brown, but antennae, tibiae, and tarsi darker. Form E (Fig. 9I): similar to form A, but apical 1/3 of elytra black.

#### Diagnosis.

*Neochya nitidissima* (Chûjô) is similar to *N. chengi* sp. nov. in having wide elytra, truncate elytral apices and reduced lateral depressions on the pronotum (Figs 4, 9) (narrow elytra, rounded elytra apices and with lateral depression on the pronotum in others (Figs 2D–F, 7) but differs from *N. chengi* sp. nov. in rounded elytra and having reduced punctures on the pronotum and fine punctures on the elytra (Fig. 9) (parallel sided elytra and coarse punctures on pronotum and elytra in *N. chengi* sp. nov. (Fig. 3)). In addition, males of both species are separated from others with smooth margin of tectum of the penis (Figs 5C, 10C) (serrate margin of tectum (Figs 8C, 12C), but males of *N. nitidissima* differs from those of *N. chengi* with small rounded process on lateral margin of the penis (Fig. 10C–E) (lacking small rounded process on lateral margin of the penis in *N. nitidissima* (Fig. 5C–E). *Atrachya bicoloripennis* (Forms A and B: Fig. 9A–E) and *A. saramao* (Form C: Fig. 9E, F) have distinct color patterns which are different from typical form *N. nitidissima* (Form D: (Fig. 9G, H)). All of they are synonyms with no doubt based on examination of the penis.

#### Remarks.

The holotype of *Luperodes nitidissimus* was described as a male (Chûjô 1935), but it is actually a female.

#### Food plants.

Celastraceae: *Celastrus kusanoi* Hayata, *C. hindsii* Benth., *Euonymus spraguei* Hayata (Fig. 1D).

#### Distribution.

**Figure 11.**
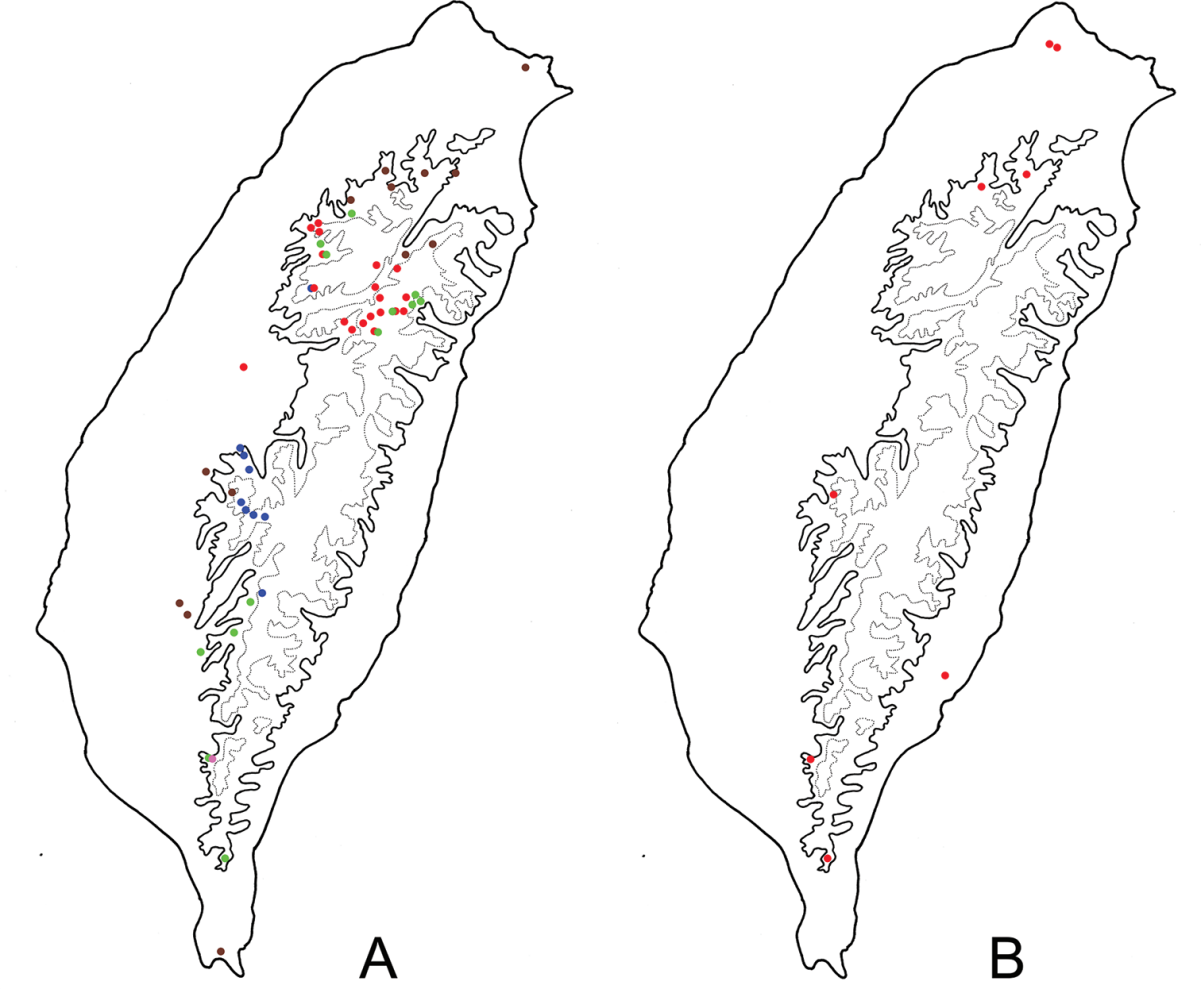
Distribution map of *Neochya* species, solid line: 1000 m, broken line: 2000 m **A***N. nitidissima* Blue Dots = Form A, red dots = form B, green dots = form C, gray dots = form D, pink dots = form E **B***N. tsoui* sp. nov.

Widespread in Taiwan. Most individuals with different color patterns can be separated based on distributions (Fig. 11A) except for the single form E. Members of form A occur at high elevation (> 2000 m) in south Taiwan, including Chiayi, south Nantou, and Kaohsiung counties. Those of form B also occur at high elevation (> 2000 m) but in central Taiwan, including Hsinchu, Miaoli, north and central Nantou, Taichung and Hualien counties. Those of form C occur at middle elevations (1000–2000 m) in central and south and east Taiwan, including Hsinchu, Miaoli, Taichung, Nantou, Kaohsiung, Pingtung, and Hualien counties. Those of form D occur at middle elevations (1000–2000 m) in north Taiwan, including Taipei, Taoyuan, Hsinchu, and Ilan counties, as well as lowlands (< 1000 m) in central and south Taiwan, including Yulin, Tainan, and Pingtung counties. Coexistence of two different color patterns was observed in some areas, such forms A and B at Anmashan (鞍馬山); forms B and C at Hsuehchien (雪見), Tayuling (大禹嶺), and Tunyuan (屯原); forms C and E at Peitawushan (北大武山).

### Neochya tsoui sp. nov.

Taxon classificationAnimaliaColeopteraChrysomelidae

FE3D2EAC-50D5-5C94-A12D-44DFB2BB1FDC

http://zoobank.org/53CBEF0B-44C9-4F4F-8912-FAE75CD2BD85

Figures 2D–F , 12

#### Types

**(*N* = 49). *Holotype*** ♂ (TARI), Taiwan. **Chiayi**: Laichitashan (來吉塔山), 19.III.2009, leg. H. Lee. ***Paratypes*.** 3♂♂, 4♀♀ (TARI), same data as holotype; **Hsinchu**: 1♂ (TARI), Lupi (魯壁), 3.II.2009, leg. H. Lee; 3♂♂, 3♀♀ (TARI), same locality, 10.III.2009, S.-F. Yu; 1♂, 1♀ (TARI), same locality, 18.IV.2009, leg. M.-H. Tsou; 1♂ (TARI), same locality, 12.VII.2009, leg. M.-H. Tsou; 3♂♂, 6♀♀ (TARI), same locality, 25.II.2010, leg. S.-F. Yu; Taipei: 1♂, 1♀ (TARI), same locality, 13.III.2011, leg. M.-H. Tsou; 3♂♂, 3♀♀ (TARI), same locality, 20.III.2011, leg. M.-H. Tsou; 1♀ (TARI), same but with “leg. S.-F. Yu”; **Pingtung**: 1♀ (TARI), Peitawushan (北大武山), 21.IX.2012, leg. J.-C. Chen; 1♀ (TARI), Tahanshan (大漢山), 22.II.2007, leg. S.-F. Yu; 1♀ (TARI), same locality, 19.VIII.2011, leg. J.-C. Chen; 4♀♀ (TARI), same locality, 2.IX.2011, leg. J.-C. Chen; 1♀ (TARI), same locality, 31.III.2012, leg. W.-C. Liao; 1♀ (TARI), same locality, 9.VI.2013, leg. Y.-T. Chung; **Taipei**: 1♂ (TARI), Fengkueitsui (風櫃嘴), 26.VII.2007, leg. M.-H. Tsou; 1♂ (TARI), Yangmingshan (陽明山), 10.III.2007, leg. M.-H. Tsou; Taitung: 1♀ (TARI), Tulanshan (都蘭山), 20.IX.2017, B.-X. Guo; **Taoyuan**: 1♂ (TARI), Lalashan (拉拉山), 14.V.2009, leg. C.-F. Lee.

#### Description.

**Figure 12.**
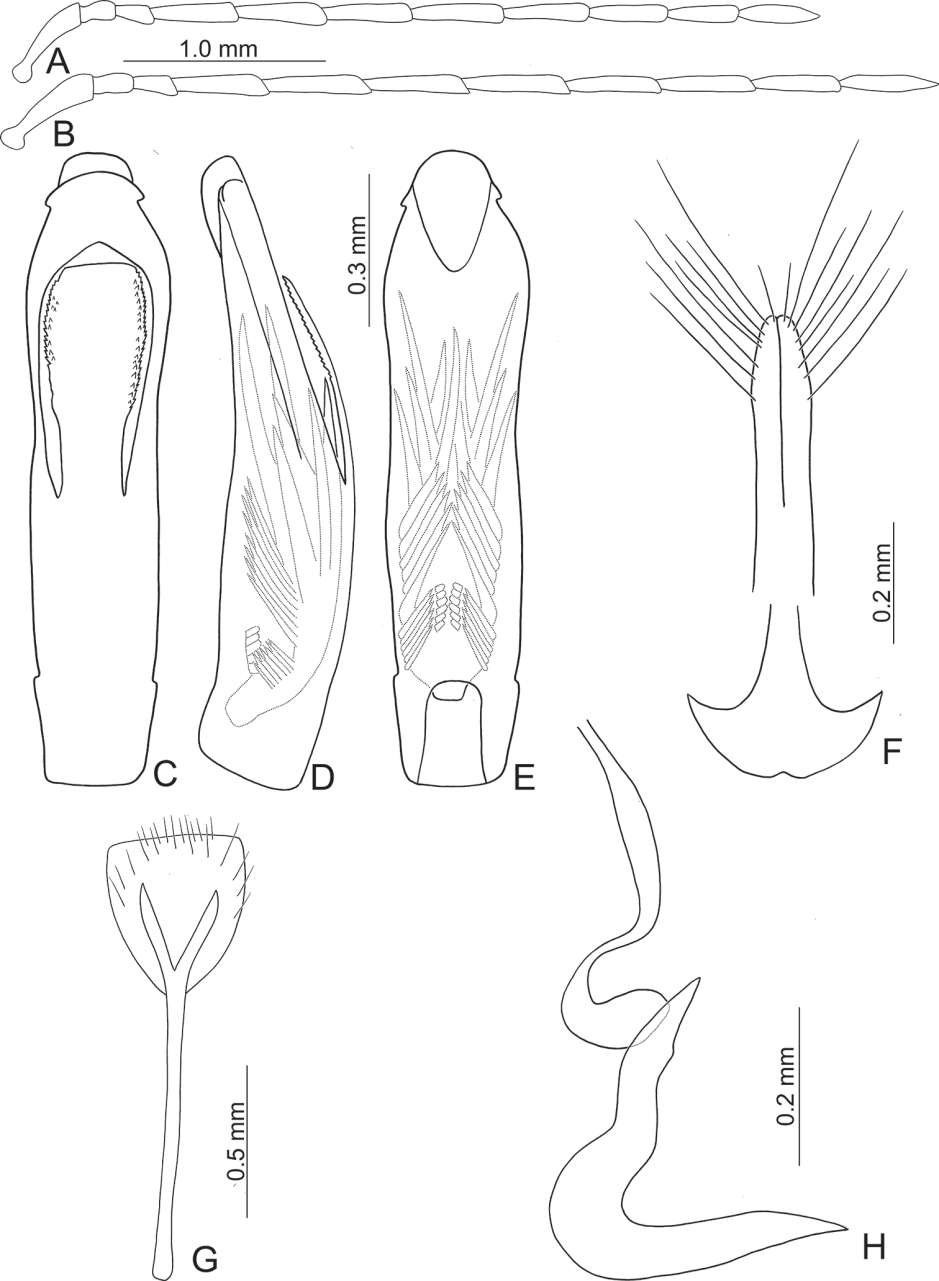
Diagnostic characters of *Neochya tsoui* sp. nov. **A** antenna, male **B** antenna, female **C** aedeagus, dorsal view **D** aedeagus, lateral view **E** aedeagus, ventral view **F** gonocoxae **G** abdominal ventrite VIII **H** spermatheca.

***Length*** 4.6–5.5 mm, width 2.1–3.0 mm. ***General color*** reddish brown or yellowish brown (Fig. 2D–F); but antennae, tibiae, and tarsi darker. ***Antennae*** (Fig. 12A) filiform in males, ratio of length of antennomeres I to XI 1.0 : 0.4 : 0.4 : 1.0 : 1.0 : 1.0 : 1.0 : 1.0 : 0.9 : 0.8 : 0.9; ratio of length to width from antennomere I to XI 3.6 : 1.7 : 1.8 : 4.5 : 5.1 : 5.3 : 5.3 : 4.7 : 4.5 : 4.1 : 4.4; a little slender in females, ratio of length of antennomeres I to XI (Fig. 12B) 1.0 : 0.4 : 0.4 : 0.9 : 1.0 : 1.0 : 1.0 : 0.9 : 0.9 : 0.8 : 0.9; ratio of length to width from antennomere I to XI 3.8 : 2.0 : 2.3 : 4.5 : 5.1 : 5.8 : 6.4 : 6.1 : 6.1 : 5.6 : 5.5. ***Pronotum*** 1.75–2.00 times wider than long; lateral margins rounded and basally narrowed, basal margin slightly rounded, apical margin slightly concave; disc with dense coarse punctures, with lateral depressions. ***Elytra*** 1.51–1.65 times longer than wide; lateral margins widest at apical 1/3; disc slightly convex, with dense, coarse punctures; apex rounded. ***Penis*** (Fig. 12C–E) wide, ca. 4.5 times longer than wide; lateral margins parallel from base to apical 2/5, then moderately broadened, widest at apical 1/5; apex broadly rounded; apical area weakly sclerotized; tectum elongate from apical 1/5 to middle, lateral margins slightly rounded and toothed, apex truncate; slightly curved in lateral view; ventral surface with membranous area from apex to apical 1/5; with one small rounded process inside lateral margin near apex. Endophallic spiculae complex with five or six pairs of hooked spiculae, with one pair of longitudinal rows of hair-like setae and small rounded sclerites near base. ***Gonocoxae*** (Fig. 12F) slender, tightly conjunct from apex to middle; each gonocoxa with eight setae from apical 1/6 to apex, subapically widened, apex truncate, base shallowly bifurcate. ***Ventrite*** VIII (Fig. 12G) weakly sclerotized except apex, with several long setae at apex, and several long setae at sides, short setae along apical margin, spiculum elongate. ***Spermathecal receptaculum*** (Fig. 12H) as slender as pump, apically tapering; pump slender and curved; sclerotized spermathecal duct extremely elongate, but base extremely wide, followed by slender tube, then with inflated areas. Bursal sclerites reduced.

#### Diagnosis.

*Neochya tsoui* sp. nov. is similar to *N. hirashimai* (Kimoto) in having slender elytra and lateral depressions on the pronotum (Figs 2D–F, 7) (wide elytra and lacking lateral depressions on the pronotum in others (Figs 4, 9), but differs from *N. hirashimai* in having reddish brown pronotum and elytra (reddish brown pronotum and yellowish brown elytra, or black pronotum, black basal half and yellowish brown apical half of elytra in *N. hirashimai*). In addition, males of both species are separated from others with serrate margin of tectum of the penis (Figs 8C, 12C) (smooth margin of tectum (Figs 5C, 10C), but males of *N. tsoui* differs from those of *N. hirashimai* with the penis widest at apical 2/5 (Fig. 12C, E) (penis widest at middle in *N. nitidissima* (Fig. 8C, E).

#### Food plants.

Celastraceae: *Euonymus japonicus* Thunb., *E. spraguei* Hayata (Fig. 1E, F).

#### Etymology.

This new species is dedicated to Mei-Hua Tsou, a member of TCRT and the first to collect this new species.

#### Distribution.

Widespread but scattered in Taiwan (Fig. 11B).

### Tsouchya gen. nov.

Taxon classificationAnimaliaColeopteraChrysomelidae

E07CCD72-0AE8-5193-A195-9BDB3D87FE60

http://zoobank.org/3DBEF74D-9D2E-40E4-BA4A-50325FBB8E01

#### Type species.

*Atrachya mediofasciata* Kimoto, 1976.

#### Description.

**Figure 13.**
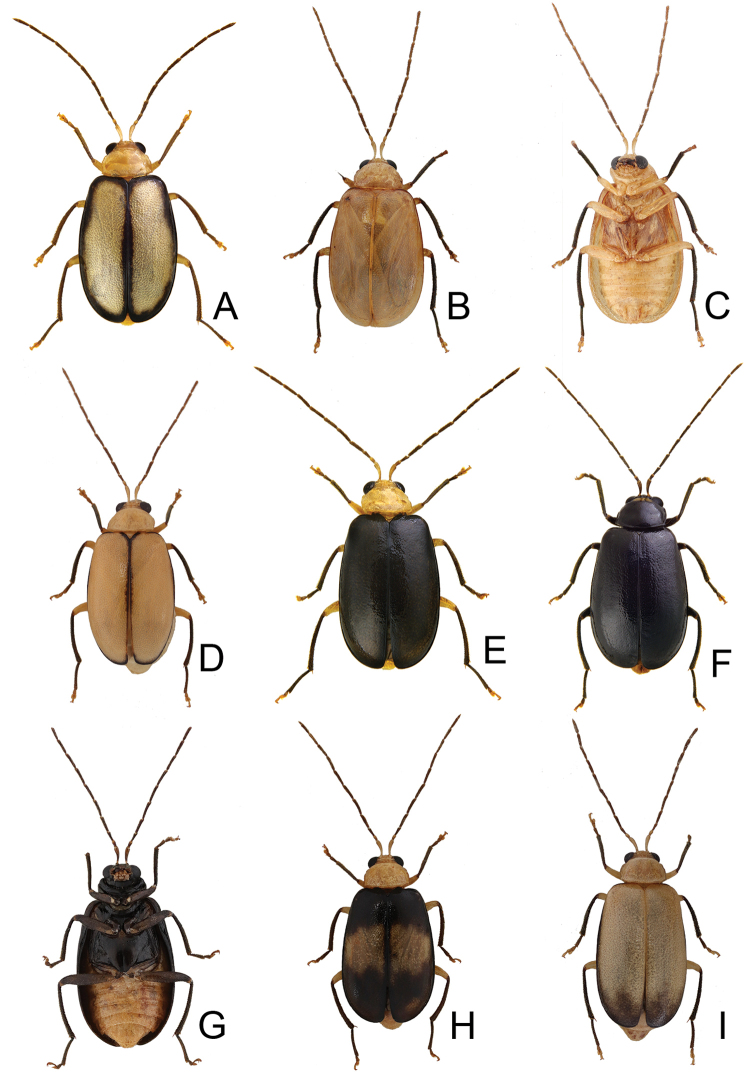
Habitus of *Tsouchya mediofasciata***A** female, from Shihshan trail (石山林道), dorsal view **B** male, from Lilungshan (里龍山), dorsal view **C** same, ventral view **D** female, from Lilungshan (里龍山), dorsal view view **E** male, from Shihshan trail (石山林道), dorsal view **F** female, from Shihshan trail (石山林道), dorsal view **G** same, dorsal view **H** male, from Talu trail (大鹿林道), dorsal view **I** female, from Meifeng (梅峰), dorsal view.

Color extremely variable (Fig. 13) but without metallic color (see remarks of *Tsouchya mediofasciata*). Body length 4.5–6.0 mm.

**Figure 14.**
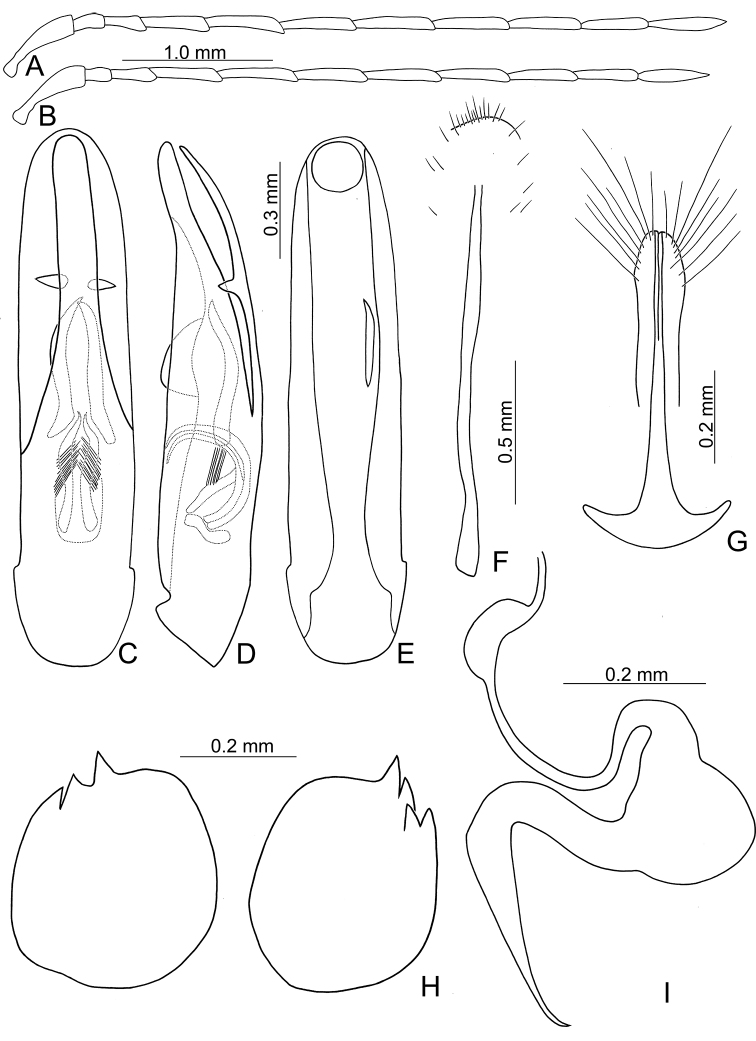
Diagnostic characters of *Tsouchya mediofasciata***A** antenna, male **B** antenna, female **C** aedeagus, dorsal view **D** aedeagus, lateral view **E** aedeagus, ventral view **F** abdominal ventrite VIII **G** gonocoxae **H** bursal sclerites **I** spermatheca.

***Head*.** Labrum trapezoidal, transverse, with six pores in transverse row bearing pale seta, anterior margin truncate. Anterior part of head short, almost impunctate and glabrous, four setae on anterior margin of clypeus and several setae along anterior margin of anterofrontal ridge. Interantennal space narrow, 1.0–1.2× as wide as diameter of antennal insertion. Frontal tubercles transverse, slightly reduced, glabrous. Vertex smooth and glabrous. Antennae slender, covered with dense setae, antennomere II much shorter than antennomere III (0.61–0.68× as long as III); similar in both sexes (Fig. 14A, B).

***Pronotum*** 1.52–1.56 times as broad as long, lateral margins straight, basally narrowed. Disc covered with dense coarse punctures, moderately convex. Posterior half of disc with wide shallow transverse impression. Anterior margin lacking marginal bead, lateral and posterior margins with marginal bead. Anterior and posterior margins without setae, lateral margins with two pairs of setae near base and apex, respectively. Anterior angles moderately swollen, rectangular, posterior angles obtuse angulate, all angles with setigerous pores bearing long pale setae.

***Scutellum*** subtriangular, impunctate, glabrous, with rounded apex.

***Elytra*** 1.55–1.61 times as long as wide, almost glabrous (with indistinct, sparse, short, pale setae on humeri, lateral margins and apical slopes), parallel-sided, densely covered with coarse confused punctures. Humeral calli well developed. Epipleura broad at base, strongly narrowed at basal 1/3, abbreviated from apical 1/3 to apex. Macropterous.

***Ventral*** surface sparsely covered with fine punctures and pale setae. Anterior coxal cavities closed (Fig. 18F). Prosternal process not visible between procoxae. Abdomen simple, posterior margin of last ventrite with two long incisions in males.

***Legs slender*.** All tibiae with one apical spine, the longest spine on metatibia. Protarsomeres I not modified in males. Metatarsomeres I much longer than pro- and mesotarsomeres I, much longer than II and III combined. Claws appendiculate.

***Penis*** (Fig. 14C–E) broad, without lateral processes; tectum elongate, apical margin truncate, with one pair of apically tapering sclerites articulated with lateral margins; internal sac with two types of endophallic spiculae (median and apical endophallic spiculae); with one rounded sclerite projecting from ventral surface.

***Gonocoxae*** (Fig. 14G) slender, tightly conjunct medially; each gonocoxa with eight setae from near apex to apical 1/6, subapically widened, apex narrowly rounded. Ventrite VIII (Fig. 14F) weakly sclerotized except apex, with several short and long setae at apex, and several long setae at sides, spiculum elongate. Spermathecal receptaculum (Fig. 14I) strongly swollen; pump slender and curved; sclerotized spermathecal duct extremely elongate, but base wide, followed by short slender tube, then with inflated areas. Bursal sclerites (Fig. 14H) paired, circular, with three or four teeth at one side.

#### Diagnosis.

*Tsouchya* gen. nov. differs from *Neochya* gen. nov., *Monolepta* Chevrolat and *Atrachya* Chevrolat based on the following combination of characters: antennomere II much short than III in length (Fig. 14A, B) (antennomere II subequal to III in *Neochya* gen. nov. (Figs 5A, B, 8A, B, 10A, B, 12A, B) and *Monolepta*); closed prothoracic coxal cavities (Fig. 18F) (open prothoracic coxal cavities in *Atrachya* (Fig. 18A) and *Neochya* gen. nov. (Fig. 18D)); absence of subscutellar impression on the elytra in males (presence of subscutellar impression on the elytra in those of *Atrachya*); penis without lateral processes near apex (Fig. 14C–E) (with lateral processes in *Neochya* gen. nov. (Figs 8C–E, 10C–E, 12C–E); tectum broad and with apical margin truncate (Fig. 14C) (tectum elongate with apex deeply bifurcate in *Atrachya* (Fig. 3C)), with one pair of lateral sclerites (Fig. 14C) (lacking lateral sclerites in others); two types of endophallic spiculae (Fig. 14C) (only one type of endophallic spiculae in *Neochya* gen. nov. (Figs 5C–E, 8C–E, 10C–E, 12C–E; three types of endophallic spiculae in *Monolepta*); spermatheca with strongly swollen receptaculum (Fig. 14I) (slender receptaculum in *Neochya* gen. nov. (Figs 5H, 8H, 10H, 12H)), without acute apex (with acute apex in *Neochya* gen. nov.); one pair of bursal sclerites (Fig. 14H) (two pairs of bursal sclerites in *Monolepta*; reduced in *Neochya* gen. nov.), circular and flattened (slender in others); ventrite VIII with few setae at sides (dense setae at sides in *Atrachya* (Fig. 3F)).

#### Etymology.

Composed from Tsou and *Atrachya* to honor Mei-Hua Tsou, who is a member of TCRT (Taiwan Chrysomelid Research Team) and made great contributions to inventorying the chrysomelid fauna in Taiwan. The gender is feminine.

#### Included species.

*Tsouchya mediofasciata* (Kimoto), comb. nov.

### Tsouchya mediofasciata

Taxon classificationAnimaliaColeopteraChrysomelidae

(Kimoto, 1976) comb. nov.

A4EF7D88-268F-5BE2-9557-57307137532D

Atrachya mediofasciata Kimoto, 1976: 6; Kimoto 1989: 257 (additional records); Kimoto 1991: 15 (additional records).
Monolepta tsoui Lee, 2009: 23. syn. nov.
Monolepta bicavipennis : Kimoto, 1969: 50 (Taiwan). non Monolepta bicavipennis Chen, 1942.

#### Types.

*Atrachy mediofasciata*. ***Holotype*** ♂ (OMNH): “[Taiwan] / Meifeng (梅峰) / Nantou Hsien [h, w] // 26.VI.1971 / Y. Miyatake [h, w] // Atrachya / mediofasciata / Kimoto, n. sp. [h, w] // HOLOTYPE [p, r] // (Redg. O.M.N.H.) [p, w]”. ***Paratypes***: 1♀ (KMNH): “(Taiwan) / Fenchihu (奮起湖), 1400m / Chiayi Hsien [p, w] // 7[h].vii.1965 / Y. Kurosawa [p, w] // Japan-U. S. / Co-op. Sci / Programme [p, y] // PARATYPE [p, b] // Atrachya / mediofasciata / Kimoto, n. sp. [h, w]”; 1♀ (KMNH): “(Taiwan) / Alishan (阿里山), 2300m / Chiayi Hsien [p, w] // 6[h].vii.1965 / Y. Kurosawa [p, w] // Japan-U. S. / Co-op. Sci / Programme [p, y] // PARATYPE [p, b] // Atrachya / mediofasciata / Kimoto, n. sp. [h, w]”.

#### Other material.

**Kaohsiung**: 1♂, 2♀♀ (KMNH), Shik Shan (石山), near Liu Kui (六龜), 9.VIII.1986, leg. K. Baba; **Nantou**: 1♂ (KMNH), Chun Yan (春陽), 7.X.1986, leg. K. Baba; 1♂ (NMNS), Juiyenhsi (瑞岩溪) Station, 29–30.VIII.2009, leg. H.-H. Liang; 1♂ (NMNS), same but with “12.VII.2007”; **Taichung**: 1♀ (NMNS), Tasheishan (大雪山) Forest Road 32km, 16.VIII.2008, leg. Liang, Chen, & Fu; 2♀♀ (BPBM), Hassenzan (= Pahsienshan, 八仙山), 23.VI.1934, leg. J. L. Gressitt, both specimens were identified as *Monolepta bicavipennis* Chen (Kimoto 1969).

#### Remarks.

This species is described in detail as *Monolepta tsoui* by Lee (2009). Color patterns of this species are extremely variable. Typical individuals have a yellowish brown body, with wide black bands along the lateral margins and suture of elytra (Fig. 13A), blackish brown antennae, except two basal antennomeres, tibiae, tarsi, metasternum, metepisternum, and epimera yellowish brown. Some have a more yellowish body color (Fig. 13B, C) but with blackish brown antenna, tibiae, and tarsi as typical form. Different degrees of variation exist between both forms, such as slender back stripes along the margin of the elytra (Fig. 13D). Some are similar to the typical form, but the elytra are entirely black (Fig. 13E). Some are entirely black except the yellowish brown abdomen (Fig. 13F, G). In addition, two color patterns have not been studied previously. One is similar to the typical form but with the elytra black except one transverse white band (Fig. 13H). It was described as *Atrachya mediofasciata*. The other is also similar to the typical form but the elytra black apically (Fig. 13I). In addition, two specimens misidentified as *Monolepta bicavipennis* have a characteristic color pattern: yellowish brown body but head and prothrax blackish brown, tibiae darker.

### Chinochya gen. nov.

Taxon classificationAnimaliaColeopteraChrysomelidae

C808AD95-0352-572D-AF73-DFCF8A4EE40A

http://zoobank.org/F1F08437-3300-4C41-B900-C765AA4E77E3

#### Type species.

*Monolepta sublata* Gressitt & Kimoto, 1963.

**Figure 15.**
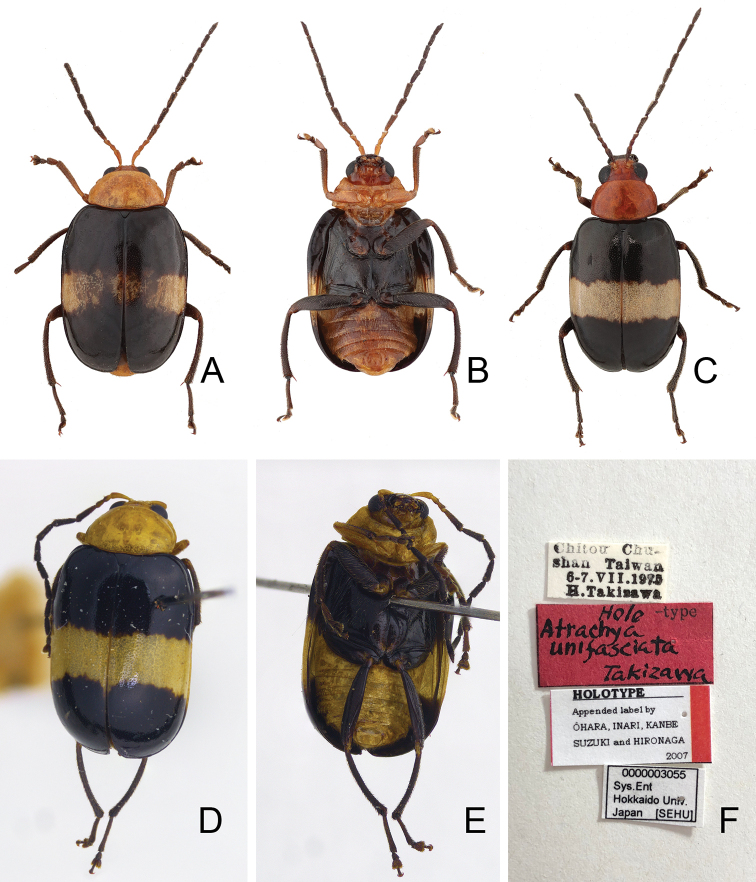
Habitus of *Chinochya sublata* and *C. unifasciata***A***C. sublata*, male, dorsal view **B** same, ventral view **C** same, female, dorsal view **D***C. unifasciata*, holotype, dorsal view **E** same, ventral view **F***C. unifasciata*, holotype, labels.

#### Description.

***Coloration*** (Fig. 15): Head, prothorax, and abdomen yellowish brown; antennae black except two basal antennomeres yellowish brown; meso- and metathoracic ventrites black; front legs yellowish brown, but tibia and tarsi darkened; middle and hind legs black; elytra black with one transverse, broad white band at middle. Body length 4.9–6.3 mm.

***Head*.** Labrum trapezoidal, transverse, with six pores in transverse row bearing pale setae, anterior margin truncate. Anterior part of head short, almost impunctate and glabrous, four setae on anterior margin of clypeus and several setae along anterior margin of anterofrontal ridge. Interantennal space narrow, 0.8–0.9× as wide as diameter of antennal insertion. Frontal tubercles transverse, slightly reduced, glabrous. Vertex smooth and glabrous. Antennae slender, covered with dense setae, antennomere II subequal to III in length; similar in both sexes.

***Pronotum*** 1.62–1.69 times as broad as long, lateral margins rounded, basally narrowed. Disc covered with dense, fine punctures, moderately convex, without transverse impression. Anterior margin lacking marginal bead, lateral and posterior margins with marginal bead. Anterior and posterior margins without setae, lateral margins with two pairs of setae near base and apex, respectively. Anterior angles moderately swollen, rectangular, posterior angles obtuse angulate, all angles with setigerous pores bearing long pale setae.

***Scutellum*** subtriangular, impunctate, glabrous, with rounded apex.

***Elytra*** 1.37–1.61 times as long as wide, almost glabrous (with indistinct, sparse, short, pale setae on humeri, lateral margins and apical slopes), parallel-sided, densely covered with coarse, confused punctures. Humeral calli well developed. Epipleura broad at base, strongly narrowed at basal 1/3, abbreviated from apical 1/3 to apex. Macropterous.

***Ventral*** surface sparsely covered with fine punctures and pale setae. Anterior coxal cavities almost closed (Fig. 18B). Prosternal process not visible between procoxae. Abdomen simple, posterior margin of last ventrite with two long incisions in males.

**Figure 16.**
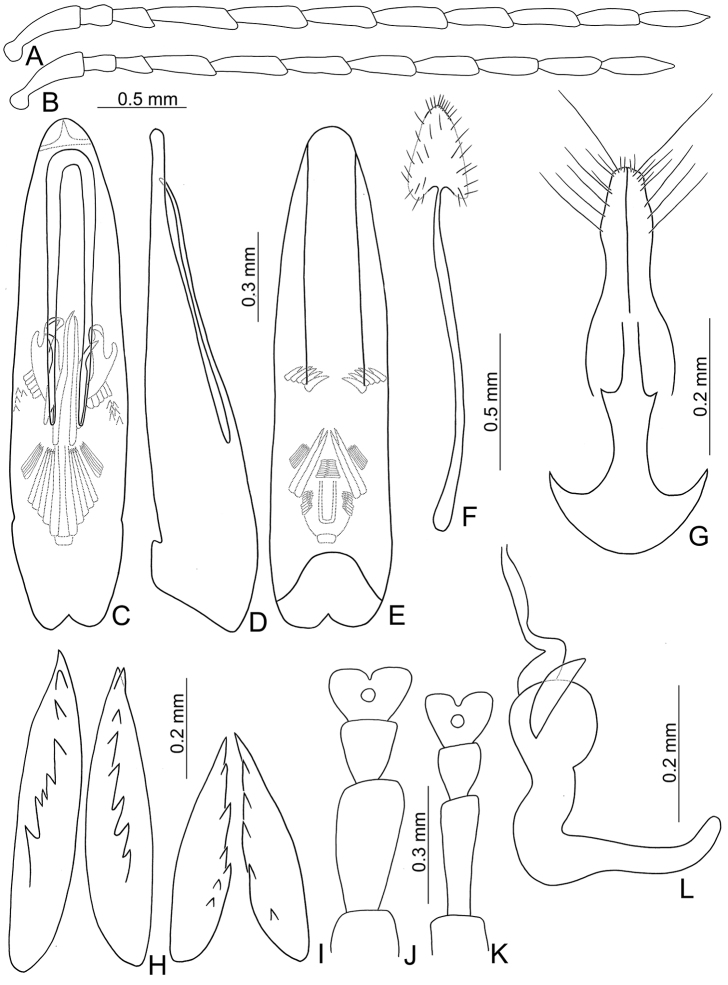
Diagnostic characters of *Chinochya sublata***A** antenna, male **B** antenna, female **C** aedeagus, dorsal view **D** aedeagus, lateral view **E** aedeagus, ventral view **F** abdominal ventrite VIII **G** gonocoxae **H** dorsal bursal sclerites **I** ventral bursal sclerites **J** protarsi, male **K** protari, female **L** spermatheca.

**Figure 17.**
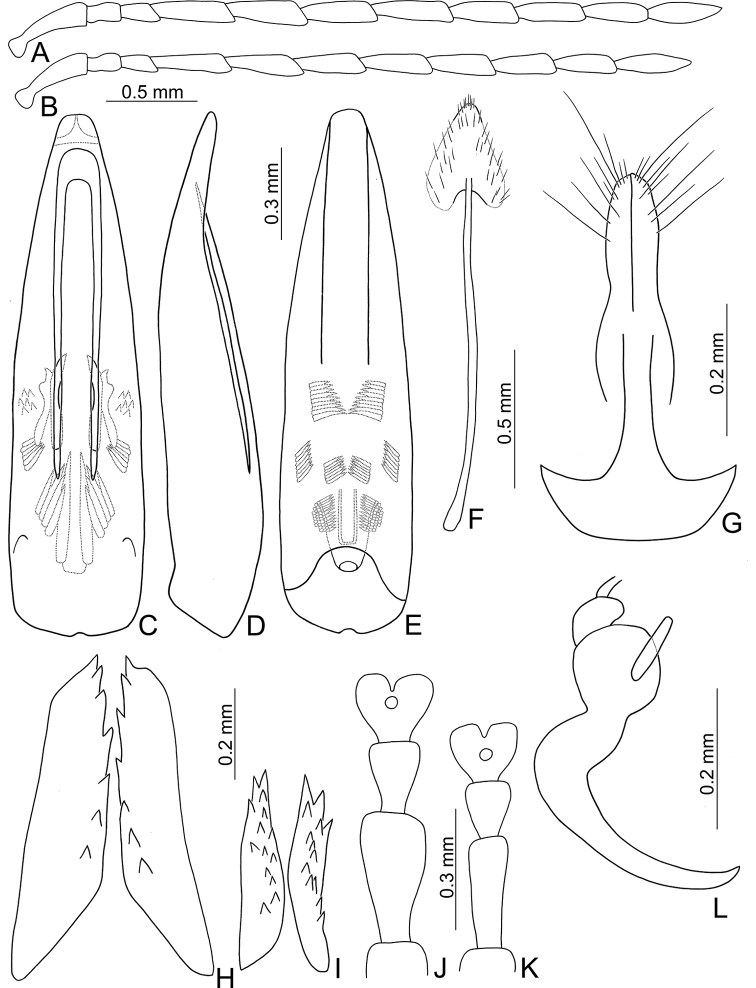
Diagnostic characters of *Chinochya unifasciata***A** antenna, male **B** antenna, female **C** aedeagus, dorsal view **D** aedeagus, lateral view **E** aedeagus, ventral view **F** abdominal ventrite VIII **G** gonocoxae **H** dorsal bursal sclerites **I** ventral bursal sclerites **J** protarsi, male **K** protari, female **L** spermatheca.

***Legs slender*.** All tibiae with one apical spine, the longest spine on metatibia. Protarsomeres I swollen in males (Figs 16J, K, 17J, K). Metatarsomeres I much longer than pro- and mesotarsomeres I, much longer than II and III combined. Claws appendiculate.

***Penis*** (Figs 16C–E, 17C–E) broad, without lateral processes; tectum elongate, apical margin truncate; internal sac with two types of endophallic spiculae (median and lateral endophallic spiculae).

***Gonocoxae*** (Figs 16G, 17G) slender, tightly conjunct medially; each gonocoxa with nine or ten setae from near apex to apex, subapically widened, apex oblique truncate. Ventrite VIII (Figs 16F, 17F) well sclerotized except apex, with several short and long setae at apex, and dense long setae at sides, spiculum elongate. Spermathecal receptaculum (Figs 16L, 17L) strongly swollen, with one erect sclerite; pump slender and curved; sclerotized spermathecal duct extremely elongate, but base wide, followed by short slender tube, then with inflated areas. Bursal sclerites (Figs 15H, I, 16H, I) with two pairs of well-developed bursal sclerites.

#### Diagnosis.

**Figure 18.**
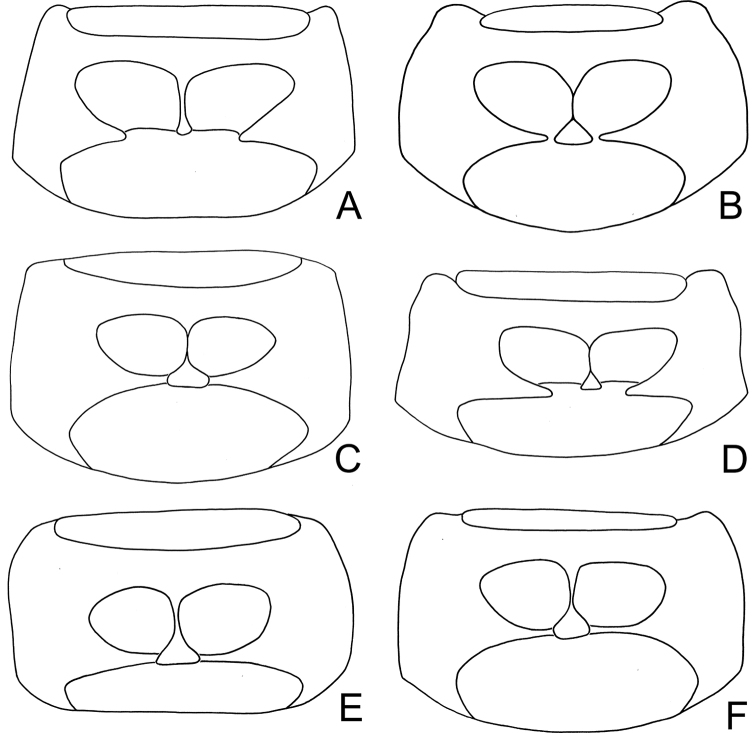
Prothorax, ventral view **A***Atrachya menetriesii***B***Chinochya unifasciata***C***Monolepta gracilipes***D***Neochya nitidissima***E***Paleosepharia formosana***F***Tsouchya mediofasciata*.

*Chinochya* gen. nov. differs from *Tsouchya* gen. nov., *Neochya* gen. nov., *Atrachya* Chevrolat, and *Monolepta* Chevrolat based on the following combination of characters: antennomere II subequal to III in length (Figs 16A, B, 17A, B) (antennomere II much shorter III in *Tsouchya* gen. nov. (Fig. 14A, B) and *Atrachya* (Fig. 3A, B)); almost closed prothoracic coxal cavities (Fig. 18B) (completely closed prothoracic coxal cavities in *Tsouchya* gen. nov. (Fig. 18F) and Taiwanese species of *Monolepta* (Fig. 18C), widely open prothoracic coxal cavities in *Atrachya* (Fig. 18A) and *Neochya* (Fig. 18D)); absence of subscutellar impression on the elytra in males (presence of subscutellar impression on the elytra in those of *Atrachya*); tarsomere I of front legs swollen in males (Figs 16J, K, 17J, K) (not modified in males of other genera); penis without lateral processes near apex (Figs 16C–E, 17C–E) (with lateral processes in *Neochya* gen. nov. (Figs 8C–E, 10C–E, 12C–E)); tectum broad and with apical margin truncate (Figs 16C, 17C) (tectum elongate with apex deeply bifurcate in *Atrachya* (Fig. 3C)), without pair of lateral sclerites (with one pair of lateral sclerites in *Tsouchya* gen. nov. (Fig. 14C)); presence of median and lateral endophallic spiculae (Figs 16C, E, 17C, E) (median and apical endophallic spiculae in *Tsouchya* gen. nov. (Fig. 14C, D); only one type of endophallic spiculae in *Neochya* gen. nov. (Figs 5C–E, 8C–E, 10C–E, 12C–E); three types of endophallic spiculae in *Monolepta*); spermatheca with strongly swollen receptaculum (Fig. 16L, 17L) (slender receptaculum in *Neochya* gen. nov. (Figs 5H, 8H, 10H, 12H)), not apically tapering (apically tapering in *Neochya* gen. nov.), with one erect sclerite (no erect sclerites in others); ventrite VIII in females with dense long setae and well sclerotized (Figs 16F, 17F) (with few setae and weakly sclerotized in others); two pairs of well-developed bursal sclerites (Figs 16H, I, 17H, I) (one pair of bursal sclerites in *Tsouchya* gen. nov. (Fig. 14H) and *Atrachya* (Fig. 3H); reduced in *Neochya* gen. nov.); nine or ten setae on each gonocoxa, some of them small (Figs 16G, 17G) (seven or eight setae on each gonocoxa, all long in others).

#### Etymology.

Composed from China and *Atrachya* to indicate the locality of the type species. The gender is feminine.

#### Included species.

*Chinochya sublata* (Gressitt & Kimoto) comb. nov. and *C. unifasciata* (Takizawa) comb. nov.

### Chinochya sublata

Taxon classificationAnimaliaColeopteraChrysomelidae

(Gressitt & Kimoto, 1963) comb. nov.

841764A4-2372-5DC4-87D1-062DBF0E8E53

Figures 15A–C , 16

Monolepta sublata Gressitt & Kimoto, 1963: 635.

#### Types.

***Paratype***: 1♀ (CAS): “Szechuan, China / NE. of Motauchi / Wanhsien, IX [p] 27 [h] 48 / 4200–4800 ft. [p, w] // Gresitt & / Djou Collrs. [p, w] // NO. 28 [p, w] // PARATYPE [P] ♀ / Monolepta / sublata [h] / Gressitt & Kimoto [p, y]”.

#### Other material.

China. Fujian: 1♀ (KMNH), Chungan, Lower Kuatun, 16.X.1941, leg. T. C. Maa; 2♂♂ (TARI), Jiuxianshan (九仙山), 21.VI.2014, leg. Y.-T. Chung; 13♂♂ (TARI), same locality, 12–17.VI.2015, leg. Y.-T. Chung; Yunnan: 3♂♂, 1♀ (TARI), Heinitang (黑泥塘), 6–9.IX.2017, leg. Y.-T. Wang; 3♂♂, 1♀ (TARI), Houqiao (猴橋), 12.VII.2016, leg. Y.-T. Wang; 1♀ (TARI), same locality, 5.IX.2018, leg. C.-C. Chen; 2♂♂ (TARI), Yunfengshan (雲峰山), 11.VII.2016, leg. Y.-T. Wang.

#### Description.

***Length*** 5.3–6.3 mm, width 3.1–3.7 mm. ***Head, prothorax, and abdomen*** yellowish brown; antennae black except two basal antennomeres yellowish brown; meso- and metathoracic ventrites black; front legs yellowish brown, but tibia and tarsi darkened; middle and hind legs black; elytra black with one transverse, broad white band at middle (Fig. 15A–C). ***Antennae*** (Fig. 16A) filiform in males, ratio of length of antennomeres I to XI 1.0 : 0.4 : 0.5 : 1.1 : 1.0 : 0.9 : 0.9 : 0.8 : 0.9 : 0.7 : 0.9; ratio of length to width from antennomere I to XI 3.8 : 1.6 : 2.0 : 4.0 : 3.6 : 3.5 : 3.3 : 3.2 : 4.0 : 4.0 : 4.3; similar in females, ratio of length of antennomeres I to XI (Fig. 16B) 1.0 : 0.4 : 0.4 : 0.8 : 0.9 : 0.8 : 0.8 : 0.8 : 0.7 : 0.7 : 0.9; ratio of length to width from antennomere I to XI 3.5 : 1.9 : 2.1 : 3.8 : 3.9 : 3.6 : 3.4 : 3.5 : 3.4 : 3.4 : 4.2. ***Pronotum*** 1.67–1.69 times wider than long; lateral margins rounded and apically narrowed, basal margin slightly rounded but slightly depressed at middle, apical margin truncate; disc with dense, fine punctures, without lateral depressions. ***Elytra*** 1.37–1.42 times longer than wide; parallel-sided; disc moderately convex, with dense, fine punctures; apex truncate. Tarsomere I of front legs swollen in males (Fig. 16J), but not modified in females (Fig. 16K). ***Penis*** (Fig. 16C–E) wide, ca. 4.5 times longer than wide; lateral margins parallel from base to apical 2/5, then basally narrowed, apex broadly rounded; with transverse and longitudinal, strongly sclerotized area near apex, intersecting at middle; tectum weakly sclerotized, elongate from apical 1/10 to basal 2/5, apex rounded; basally broadened in lateral view; ventral surface with broad groove from apex to middle. Endophallic spiculae complex: median endophallic spiculae composed of three pairs of different shapes, one pair elongate and with acute apices near middle, another pair hook-like between inner and outer pairs, the last pair bifurcate at middle; lateral endophallic spiculae composed of transverse row of hook-like, larger setae ventrally located, small setae dorsally located. ***Gonocoxae*** (Fig. 16G) slender, tightly conjunct from apex to apical 2/5; each gonocoxa with ten setae from apical 1/5 to apex, some setae very small; subapically widened; apex oblique truncate. ***Ventrite*** VIII (Fig. 16F) strongly sclerotized except apex, with a number of long setae at sides, short setae along apical margin, spiculum elongate. ***Spermathecal receptaculum*** (Fig. 16L) strongly swollen, with one transverse, erect sclerite; pump slender and curved; sclerotized spermathecal duct extremely elongate, but base extremely wide, followed by short slender tube, then with inflated areas. Bursal sclerites paired and well developed, dorsal bursal sclerites larger (Fig. 16H), with one longitudinal row of eight stout setae; ventral bursal sclerites smaller (Fig. 16I), with one longitudinal row of seven or eight small denticles.

#### Diagnosis.

*Chinochya sublata* is similar to *C. unifasciata*. They cannot be separated based on their external morphology, however, *C. sublata* (Fig. 16C–E) differs from *C. unifasciata* (Fig. 17C–E) in genitalic characters as follow: median endophallic spiculae composed of three different pairs of sclerites (only two pairs of sclerites in *C. unifasciata*); lateral endophallic spiculae transversely arranged (longitudinally arranged in *C. unifasciata*); ventral bursa sclerite with seven or eight small denticles (13 or 14 small denticles in *C. unifasciata*).

#### Remarks.

Males are here described for the first time. Types on which the original description was based are all females (Gressitt and Kimoto 1963)

#### Distribution.

South China (Fujian, Sichuan, Yunnan).

### Chinochya unifasciata

Taxon classificationAnimaliaColeopteraChrysomelidae

(Takizawa, 1978) comb. nov.

39E3469F-8B54-52B1-B579-C927ACD313B5

Figures 15D–F , 17

Atrachya unifasciata Takizawa, 1978: 132.
Monolepta sublata : Kimoto, 1976: 6 (Taiwan). non Monolepta sublata Gressitt & Kimoto, 1963

#### Types.

***Holotype*** ♂ (SEHU) (Fig. 15D–F): “Chitou (溪頭) Chu- / shan Taiwan / 6–7.VII.1975 / H. Takizawa [p, w] // Holo [h] -type [p] / Atrachya / unifasciata / Takizawa [h, r] // **HOLOTYPE** / Appended label by ÔHARA, IMRAI, KANBE / SUZUKI and HIRONAGA / 2007 [p, w, with red band along right margin] // 0000003055 / Sys. Ent / Hokkaido Univ. / Japan [SEHU] [p, w]”.

#### Other material.

Taiwan. **Hsinchu**: 1♂ (TARI), Talu trail (大鹿林道), 24.VI.2009, leg. Y.-F. Hsu; **Maioli**: 2♂♂ (TARI), Hsuehchien (雪見), 7.VI.2013, leg. W.-B. Yeh; **Kaohsiung**: 2♂♂ (TARI), Chungchihkuan (中之觀), 3.VII.2009, leg. S.-F. Yu & M.-H. Tsou; **Taitung**: 1♀ (TARI), Liyuan (栗園), 19.VI.2013, leg. B.-X. Guo.

#### Description.

***Length*** 4.9–5.8 mm, width 2.6–3.0 mm. ***Head, prothorax, and abdomen*** yellowish brown; antennae black except two basal antennomeres yellowish brown; meso- and metathoracic ventrites black; front legs yellowish brown, but tibiae and tarsi darker; middle and hind legs black; elytra black with one transverse, broad, white band at middle (Fig. 15D, E). ***Antennae*** (Fig. 16A) filiform in males, ratio of length of antennomeres I to XI 1.0 : 0.4 : 0.4 : 0.9 : 0.9 : 0.9 : 0.9 : 0.8 : 0.8 : 0.7 : 0.9; ratio of length to width from antennomere I to XI 3.8 : 1.8 : 2.0 : 3.9 : 3.4 : 3.5 : 3.6 : 3.4 : 3.6 : 3.3 : 3.5; similar in females, ratio of length of antennomeres I to XI (Fig. 17B) 1.0 : 0.4 : 0.5 : 1.0 : 1.0 : 0.9 : 0.9 : 0.9 : 0.8 : 0.8 : 0.9; ratio of length to width from antennomere I to XI 3.8 : 1.8 : 2.1 : 4.2 : 3.6 : 3.3 : 3.6 : 3.4 : 3.3 : 3.4 : 4.2. ***Pronotum*** 1.62–1.67 times wider than long; lateral margins rounded and apically narrowed, basal margin slightly rounded but slightly depressed at middle, apical margin truncate; disc with dense fine punctures, without lateral depressions. ***Elytra*** 1.51–1.61 times longer than wide; parallel-sided; disc moderately convex, with dense, fine punctures; apex truncate. Tarsomeres I of front legs swollen in males (Fig. 17J), but not modified in females (Fig. 17K). ***Penis*** (Fig. 17C–E) wide, ca. 4.0 times longer than wide; lateral margins parallel from base to apical 2/5, then basally narrowed, apex broadly rounded; with transverse and longitudinal, strongly sclerotized area near apex, intersecting at middle; tectum weakly sclerotized, elongate from apical 1/8 to basal 1/3, apex rounded; basally broadened in lateral view; ventral surface with broad groove from apex to middle. Endophallic spiculae complex: median endophallic spiculae composed with two pairs of different shapes, one pair elongate and with acute apices near middle, outer pair hook-like; lateral endophallic spiculae composed of longitudinal row of hook-like, larger setae ventrally located, small setae dorsally located. ***Gonocoxae*** (Fig. 17G) slender, tightly conjunct from apex to apical 2/5; each gonocoxa with nine setae from apical 1/5 to apex, some setae smaller, subapically widened, apex obliquely truncate. ***Ventrite*** VIII (Fig. 17F) strongly sclerotized except apex, with a number of long setae at sides, short setae along apical margin, spiculum elongate. S***permathecal receptaculum*** (Fig. 17L) strongly swollen, with one transverse, erect sclerite; pump slender and curved; sclerotized spermathecal duct short (broken), but base extremely wide. Bursal sclerites well developed, dorsal bursal sclerites larger (Fig. 17H), with one longitudinal row of seven stout setae; the ventral bursal sclerites smaller (Fig. 17I), with one longitudinal row of 13 or 14 small denticles.

#### Diagnosis.

*Chinochya unifasciata* is similar to *C. sublata*. They cannot be separated based on their external morphology, however, *C. unifasciata* (Fig. 17C–E) differs from *C. sublata* (Fig. 16C–E) based on genitalic characters as follow: median endophallic spiculae composed of two pairs of sclerites (three different pairs of sclerites in *C. sublata*); lateral endophallic spiculae longitudinally arranged (transversely arranged in *C. sublata*); ventral bursa sclerite with 13 or 14 small denticles (seven or eight small denticles in *C. sublata*).

#### Distribution.

Widespread but scattered in Taiwan.

**Figure 19.**
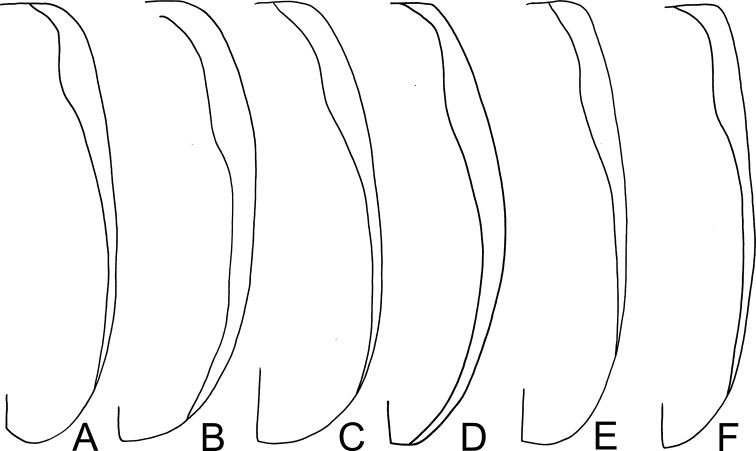
Elytron, ventral view **A***Atrachya menetriesii***B***Chinochya unifasciata***C***Monolepta gracilipes***D***Neochya nitidissima***E***Paleosepharia formosana***F***Tsouchya mediofasciata*.

### Key to genera of Monoleptites with elongate metatarsomere I and species of *Neochya* gen. nov. in Taiwan

**Table:** 

1	Subscutellar groove on the elytra present in males	***Paleosepharia* Laboissière**
–	Subscutellar groove on the elytra absent in males	**2**
2	Tarsomere I of front legs swollen in males (Fig. 16J, K; 17J, K)	***Chinochya* gen. nov.**
–	Tarsomere I not modified in males	**3**
3	Antennomere III longer than II	***Tsouchya* gen. nov.**
–	Antennomere III subequal or smaller than II	**4**
4	Prothoracic coxal cavities closed (Fig. 18C)	***Monolepta* Chevrolat**
–	Prothoracic coxal cavities open (Fig. 18D)	***Neochya* gen. nov. 5**
5	Elytra slender (Figs 2D–F, 4), 1.5–1.7× longer than wide, with apices rounded	**6**
–	Elytra wide (Figs 4, 9), 1.3–1.4× longer than wide, with apices truncate	**7**
6	Pronotum and elytra reddish brown (Fig. 2D–F)	***N. tsoui* sp. nov.**
–	Pronotum reddish brown and elytra yellowish brown; or pronotum black and elytra with basal half black and apical half yellowish brown (Fig. 7)	***N. hirashimai* (Kimoto)**
7	Elytra parallel sided; punctures on pronotum and elytra coarse	***N. chengi* sp. nov.**
–	Elytra with lateral margins rounded; punctures on pronotum reduced and punctures on elytra fine	***N. nitidissima* (Chûjô)**

## Discussion

A number of genera of Monoleptites in Sundaland with elongate metatarsomeres I were reevaluated and redefined recently, together with new genera, including *Arcastes* Baly, 1865 (Hazmi and Wagner 2010c), *Luperodes* Motschulsky, 1858 (Wagner and Bieneck 2012), *Neolepta* Jacoby, 1884 (Hazmi and Wagner 2013), *Ochralea* Clark, 1865 (Hazmi and Wagner 2010a), *Orthoneolepta* Hazmi & Wagner, 2013, *Paraneolepta* Hazmi & Wagner, 2013, *Rubrarcastes* Hazmi & Wagner, 2010a. This high supraspecific diversity occurs in both Oriental and African regions (Wagner 2007). However, no endemic genera were known in the eastern Palaearctic region, which includes Taiwan. The present study revealed that high supraspecific diversity may also occur in this area when more species of *Monolepta* and *Atrachya* are studied in detail. In addition, a number of genera appear in mainland China, Vietnam, Laos, and Thailand, including *Macrima* Baly, 1878, *Pseudosepharia* Laboissière, 1936, and *Desbordelepta* Nguyen & Gómez-Zurita, 2017. They are awaiting redescription and comprehensive revision.

Reliable diagnostic characters for the supraspecific taxonomy of Monoleptites with elongate metatarsomeres I have been limited. Color patterns are useful for most Oriental genera, but not diagnostic for east Palaearctic genera. This character in most species of *Neochya* and *Tsouchya* is variable, and in *N. nitidissima* and *T. mediofasciata* it is extremely variable. Although color patterns of *Chinochya* species are similar, some species of *Monolepta* share these patterns, including *M. leechi* Jacoby, 1890 and *M. maana* Gressitt & Kimoto, 1963.

Prothoracic coxal cavities have been used for diagnosis of genera within this group of Monoleptites. This character was evaluated in *Atrachya menetriesii*, all species of *Neochya*, *Chinochya*, *Tsouchya*, Taiwanese species of *Paleosepharia*, and *Monolepta*. It can be separated into three states: widely open for *Atrachya* (Fig. 18A) and *Neochya* (Fig. 18D), almost closed for *Chinochya* (Fig. 18B), completely closed for *Monolepta* (Fig. 18C), *Paleosepharia* (Fig. 18E), and *Tsouchya* (Fig. 18F).

Elytral epipleurae of *Paleosepharia* (Fig. 19C) are abbreviated before the middle and this has been considered diagnostic (e.g., Lee 2018). However, interpreting subtle differences in character states is difficult when comparing it in other related genera (Fig. 19). It is not diagnostic for distinguishing *Paleosepharia* from others. In addition, few female genitalic characters have been used as diagnostic characters. Spermathecae are poorly illustrated, and abdominal ventrites VIII and gonocoxae were usually ignored in most papers. This study supports the use of these characters as diagnostic provided that descriptions are supported by good quality illustrations.

## Supplementary Material

XML Treatment for Atrachya

XML Treatment for Neochya

XML Treatment for Neochya chengi

XML Treatment for Neochya hirashimai

XML Treatment for Neochya nitidissima

XML Treatment for Neochya tsoui

XML Treatment for Tsouchya

XML Treatment for Tsouchya mediofasciata

XML Treatment for Chinochya

XML Treatment for Chinochya sublata

XML Treatment for Chinochya unifasciata
